# Electrophysiological and Contractile Effects of Disopyramide in Patients With Obstructive Hypertrophic Cardiomyopathy: A Translational Study

**DOI:** 10.1016/j.jacbts.2019.06.004

**Published:** 2019-10-09

**Authors:** Raffaele Coppini, Cecilia Ferrantini, Josè Manuel Pioner, Lorenzo Santini, Zhinuo J. Wang, Chiara Palandri, Marina Scardigli, Giulia Vitale, Leonardo Sacconi, Pierluigi Stefàno, Laura Flink, Katherine Riedy, Francesco Saverio Pavone, Elisabetta Cerbai, Corrado Poggesi, Alessandro Mugelli, Alfonso Bueno-Orovio, Iacopo Olivotto, Mark V. Sherrid

**Affiliations:** a Department NeuroFarBa, University of Florence, Florence, Italy; b Department of Experimental and Clinical Medicine, University of Florence, Florence, Italy; c Cardiomyopathy Unit, Careggi University Hospital, Florence, Italy; d Department of Computer Sciences, University of Oxford, Oxford, United Kingdom; e European Laboratory for Nonlinear Spectroscopy (LENS), University of Florence, Sesto Fiorentino, Italy and National Institute of Optics, National Research Council, Florence, Italy; f Division of Cardiology, San Francisco Veterans Affairs Medical Center and University of California-San Francisco, San Francisco, California; g Hypertrophic Cardiomyopathy Program, New York University Langone Health, New York, New York

**Keywords:** action potentials, arrhythmias, diastolic dysfunction, hypertrophic cardiomyopathy, QT interval, safety, AP, action potential, DAD, delayed afterdepolarization, EAD, early afterdepolarization, ECG, electrocardiography, hERG, human ether-à-go-go-related gene, HCM, hypertrophic cardiomyopathy, I_Ca-L_, L-type Ca current, I_K_, delayed-rectifier K current, I_NaL_, late Na current, LVOT, left ventricular outflow tract, NCX, Na^+^/Ca^2+^ exchanger, pCa, Ca activation level, RyR, ryanodine receptor, SR, sarcoplasmic reticulum

## Abstract

Disopyramide is effective and safe in patients with obstructive hypertrophic cardiomyopathy. However, its cellular and molecular mechanisms of action are unknown. We tested disopyramide in cardiomyocytes from the septum of surgical myectomy patients: disopyramide inhibits multiple ion channels, leading to lower Ca transients and force, and shortens action potentials, thus reducing cellular arrhythmias. The electrophysiological profile of disopyramide explains the efficient reduction of outflow gradients but also the limited prolongation of the QT interval and the absence of arrhythmic side effects observed in 39 disopyramide-treated patients. In conclusion, our results support the idea that disopyramide is safe for outpatient use in obstructive patients.

## Highlights

• In patients with HCM and symptomatic LVOT-obstruction, first treatment with disopyramide leads to a marked reduction of LVOT gradients, with a slight decrease of resting ejection fraction and a modest increase of corrected QT interval, highlighting high efficacy and safety.
• In single cardiomyocytes and intact trabeculae from surgical samples of patients with obstructive HCM, in vitro treatment with 5 μmol/l disopyramide lowered force and Ca2^+^ transients while reducing action potential duration and the rate of arrhythmic afterdepolarizations.
• These effects are mediated by the combined inhibition of peak and late Na^+^ currents, L-type Ca^2+^ current, delayed-rectifier K^+^ current, and ryanodine receptors.
• In addition to the negative inotropic effect of disopyramide, in vitro results suggest additional antiarrhythmic actions.

## Visual Abstract

**Figure:**
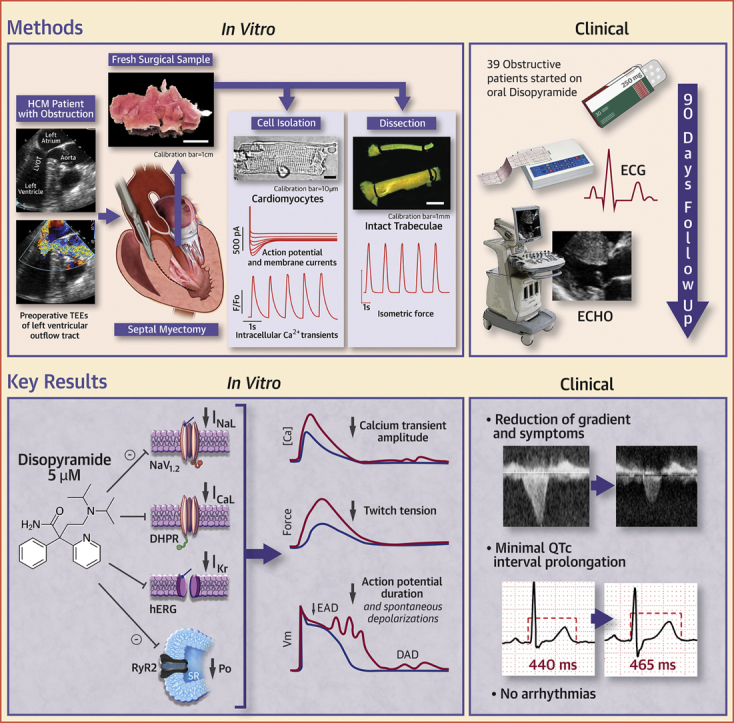

Disopyramide is a potent negative inotrope (1). It has been used to decrease left ventricular outflow tract (LVOT) obstruction in obstructive hypertrophic cardiomyopathy (HCM) since the first reports in the early 1980s and after confirmatory studies of its efficacy and safety 2, 3, 4, 5, 6. In patients with obstructive HCM and limiting symptoms, disopyramide in addition to a beta blocker has a Class I recommendation by the 2014 European Society of Cardiology guidelines and a Class IIa recommendation by the 2011 American Heart Association/American College of Cardiology Foundation guidelines 7, 8. Most recently, safe initiation in outpatients has been retrospectively demonstrated (9). Its practical use and current place in the armamentarium for obstructive HCM has been described and reviewed elsewhere 10, 11.

Despite its active use since the 1980s, there has been little work on the intracellular mechanism of therapeutic effects of disopyramide beyond its categorization as a type Ia antiarrhythmic, that is, as Na channel blocker with action potential (AP)-prolonging effects 12, 13. A paper in the late 1980s suggested an effect on sarcolemmal Ca influx and efflux mediated by the Na^+^/Ca^2+^ exchanger (NCX) (14). In light of its potent negative inotropic effects, it is not known whether the drug has additional direct effects on Ca^2+^ current, Ca^2+^ release from the sarcoplasmic reticulum (SR) or on the actin-myosin interaction and the effects of disopyramide in human HCM cardiomyocytes have not been characterized.

We previously analyzed the electromechanical profile of cardiomyocytes isolated from myectomy samples of patients with obstructive HCM 15, 16. When compared with control cells, HCM cardiomyocytes showed prolonged AP, frequent afterdepolarizations, slower Ca^2+^ transients, and elevated diastolic Ca^2+^ concentration, largely determined by overexpression of the late Na^+^ current (I_NaL_). Indeed, these electromechanical abnormalities were reversed by the I_NaL_ inhibitor ranolazine, with beneficial effects on diastolic function and cellular arrhythmias 15, 16.

In the present study, we sought to uncover mechanistic insights by applying electrophysiological and biophysical techniques to evaluate the effects of disopyramide on ion fluxes, afterdepolarizations, and twitch tension in isolated HCM cardiomyocytes and intact trabeculae harvested from patients undergoing surgical septal myectomy. In a translational approach, the in vitro study was combined with the first prospective characterization of the electrocardiographic and echocardiographic changes in patients with obstructive HCM started on disopyramide treatment.

## Methods

Detailed methods are available in the Supplemental Appendix data supplement.

### Prospective study of patients with obstructive HCM treated with disopyramide

From October 1, 2015, to January 31, 2018, 39 patients with limiting heart failure symptoms and elevated LVOT gradients were initiated on sustained release disopyramide at New York University Langone Medical Center as previously described 4, 5, 6, 9. Initial disopyramide dosage was 250 mg twice a day, followed by drug titration up to a maximum dosage of 300 mg every 12 h. Electrocardiography (ECG) was performed at each visit by recording 3 12-lead 15-s strips (collected at least 5 min apart), and QTc interval was calculated from each strip by averaging the QT intervals measured by a blinded operator from the 12 leads and correcting them for heart rate using the Bazett and the Fredericia methods. QTc values from the 3 repeated ECG strips were then averaged to obtain the final value. Echocardiography was performed to measure transmitral flow velocities and mitral annular diastolic velocities and to calculate resting LVOT gradients and LV ejection fraction before and after disopyramide.

### Patients for studies in cells and trabeculae

In vitro studies were performed at the University of Florence. Protocols were approved by the ethical committee of Careggi University Hospital (2006/0024713, renewed May 2009). We enrolled 20 patients with HCM regularly followed by our Cardiomyopathy Unit and consecutively referred to surgical myectomy for relief of drug-refractory symptoms related to LVOT obstruction. Among the 20 patients, 12 agreed to undergo mutational screening in sarcomeric genes. Clinical and genetic data are found in Supplemental Table 1.

### Statistics (Clinical studies)

Clinical data from patients are expressed as mean ± SD. Comparisons of clinical variables before and after disopyramide were performed using Student’s paired *t*-tests. To assess the relationship between the initial QTc and the increase in QTc with disopyramide, we calculated the Pearson correlation coefficient (*r*) and its significance level using MedCalc version 19.0 (MedCalc Software, Ostend, Belgium).

### Tissue processing and cell isolation

Septal specimens from 20 consecutive surgical patients were collected from the surgeon and immediately washed with cardioplegic solution and processed within 30 min from excision. Each sample was given a unique anonymous identification number (ID 1 to ID 20). Endocardial trabeculae suitable for mechanical measurements were dissected and the remaining tissue was minced and subjected to enzymatic dissociation to obtain viable single myocytes, as previously described (17).

### Single cell studies

A perforated patch whole-cell current clamp was used to measure membrane potential, as previously described (15). [Ca^2+^] variations were simultaneously monitored using the Ca^2+^-sensitive fluorescent dye FluoForte (Enzo Life Sciences, Farmingdale, New York). A whole-cell ruptured patch voltage clamp was used to record peak and late Na^+^ current, L-type Ca^2+^ current (I_Ca-L_), and delayed rectifier K^+^ current (I_K_), using appropriate protocols and solutions (15).

### Intact trabeculae studies

Ventricular trabeculae were mounted between a force transducer and a motor for muscle length control (15), and isometric force was recorded under different stimulation protocols. In brief, we evaluated the inotropic responses to increased pacing frequencies and the kinetics of isometric twitches. Resting sarcomere length was 1.9 ± 0.1 μm.

### Demembranated trabeculae

Ventricular trabeculae were skinned by exposure to 0.5% Triton X100 solution. Demembranated trabeculae were employed to obtain calcium concentration (pCa)-tension curves as previously described 15, 18, 19. Sarcomere energetics was assessed in demembranated trabeculae by simultaneous measurement of isometric force and adenosine triphosphatase activity with an enzyme-coupled assay 15, 18, 19; measurements were repeated in the presence of disopyramide.

### Drug studies

For experiments on isolated cardiomyocytes and trabeculae, disopyramide was used at the concentration of 5 μmol/l, unless otherwise specified. Test recordings in presence of the drug were performed after >3 min from the beginning of drug exposure. Afterward, the drug was washed out for >5 min and measurements were repeated. Of note, all the effects of disopyramide in isolated cardiomyocytes and trabeculae disappeared after 5 min of drug washout.

### Ca^2+^ sparks in permeabilized myocytes

Ventricular myocytes were isolated from the hearts of 4 transgenic HCM mice carrying the R92Q troponin-T mutation, as previously described 18, 19. Myocytes were permeabilized with saponin (20) and resuspended in an intracellular buffer containing 150 nmol/l free [Ca^2+^] and 5 μmol/l of the Ca^2+^-sensitive dye Asante Na^+^-green K^+^-salt (Teflabs, Austin, Texas). The frequency of spontaneous Ca^2+^ sparks was evaluated with a confocal microscope through line scan along the longitudinal cell axis (20). Myocytes incubated with vehicle were compared with cells exposed to 5 μmol/l of disopyramide. The rate of sparks was calculated from confocal line-scan recordings using the SparkMaster ImageJ plugin (National Institutes of Health, Bethesda, Maryland) for automated analysis (21).

### Statistics (Studies on cells and trabeculae)

None of the 20 consecutively collected patient samples was excluded from the final analysis. However, we were unable to perform all the different experimental procedures described herein in all 20 samples. Therefore, each dataset comprises results from cells or trabeculae isolated from 3 to 11 patient samples. For each dataset, we indicated in the respective figure legends, the total number of cells/trabeculae included, as well as the number (and ID) of patients’ samples from which they were isolated. Results from each dataset are expressed as mean ± SEM. Statistical analysis, taking into account non-Gaussian distribution, inequality of variances and within-subject correlation, was performed as previously described 15, 18. In brief, to reduce the risk of type I errors resulting from the stronger interrelationship among cells/trabeculae isolated from the same patient sample, we used hierarchical statistics including 2 nested levels (patients and cells/trabeculae) (22), plus a third hierarchical level (presence or absence of disopyramide in the same cell/trabecula) to assess the effects of drug treatment in a pairwise fashion. This approach was implemented using linear mixed models in Stata 12.0 (StataCorp LLC, College Station, Texas). The p values were calculated using linear-mixed models. For categorical data (e.g., occurrence of cellular arrhythmias), we used the Fisher exact test. We considered p < 0.05 statistically significant.

### Modeling studies

Cellular mechanisms of disopyramide action on dispersion of repolarization were investigated in a population of human ventricular cardiomyocyte models (n = 250) in control versus HCM remodeling, calibrated against human AP and Ca^2+^-transient data, as previously described (23). Unpaired or paired *t* tests, as well as linear fitting with r-squared calculation, were employed to analyze data from populations of models. Mechanisms of dispersion at the whole ventricular level were investigated in a cardiac magnetic resonance–based anatomical model of a patient with obstructive HCM, under realistic human activation sequence and heterogeneity in repolarizing currents (24).

## Results

### Prospective study of patients with obstructive HCM started on disopyramide

Thirty-nine patients were begun on disopyramide (age 66 ± 10 years; 59% were female). The mean daily dosage of disopyramide after 3 months was 497 ± 87 mg/day. No patient had clinically significant arrhythmia or organ toxicity during the study period. In 9 patients (23%), disopyramide was ineffective or there were limiting vagolytic side effects that led to termination of drug before 3 months.

#### Electrocardiograms After Disopyramide

ECG parameters before and at a median of 96 days after disopyramide initiation are shown in Table 1. There were prolongations in the QTc, QRS, JT, and PR intervals, but no significant change in heart rate at 3 months. The mean QTc interval increased from 458 ± 22 ms to 486 ± 27 ms (p < 0.001). Mean QTc prolongation was 27 ms, which corresponds to a 5.8% increase from baseline. Patients with an initially longer QTc interval (greater than the median 457 ms) had a smaller increase in QTc interval than did patients with shorter initial QTc interval (ΔQTc: 18 ± 4 ms vs. 34 ± 8 ms, p = 0.007). There was an inverse correlation between the initial QTc interval and the increase in QTc interval with disopyramide (Pearson r = −0.44, p = 0.008).

**Table 1:** Clinical Study: ECG (N = 39)

Interval (ms)	JT	QTc Interval (Bazett)	QTc Interval (Fredericia)	QRS Complex	PR	HR
Pre-disopyramide	354 ± 24	458 ± 22	453 ± 20	103 ± 19	179 ± 39	64 ± 12
4 days	368 ± 30	484 ± 34	485 ± 31	113 ± 23	200 ± 36	59 ± 11
% change	+4 ± 2	+5 ± 2	+6 ± 2	+9 ± 5	+10 ± 7	−8 ± 3
p value vs. pre-disopyramide	<0.002	<0.001	<0.001	<0.001	<0.001	0.022
23 days	366 ± 30	476 ± 37	479 ± 34	109 ± 26	191 ± 32	58 ± 10
% change	+3 ± 1	+4 ± 2	+5 ± 2	+6 ± 3	+6 ± 4	−10 ± 3
p value vs. pre-disopyramide	0.002	<0.001	<0.001	0.015	<0.001	0.002
96 days	375 ± 35	486 ± 27	485 ± 24	112 ± 23	190 ± 33	61 ± 11
% change	+6 ± 2	+6 ± 3	+7 ± 3	+8 ± 3	+6 ± 4	−5 ± 2
p value vs. pre- disopyramide	<0.001	<0.001	<0.001	<0.001	0.015	0.136

Values are mean ± SD. The p values were calculated using paired Student’s *t*-test. Electrocardiographic (ECG) intervals were measured before disopyramide and after different periods from drug initiation (4, 23, 96 days), in 39 obstructive hypertrophic cardiomyopathy patients. QTc was determined using Bazett formula or Fredericia formula.

HR = heart rate; JT = time from the end of the QRS complex to the end of the T wave; PR = time from the beginning the P wave to the beginning of the QRS complex.

#### Echocardiography

Initial resting LVOT gradients were 58 ± 49 mm Hg. After 96 days of disopyramide, gradients were reduced to 25 ± 26 mm Hg (p < 0.001). There were no differences in tissue Doppler variables, transmitral flow velocities, and in the calculations from tissue Doppler and transmitral flow velocities used to estimate left atrial pressure (Table 2). Ejection fraction was minimally reduced by disopyramide from 72.2% to 68%, an average decrease by 5.8 ± 3.1% (Table 2).

**Table 2:** Clinical Study: Echocardiography (N = 39)

	Doppler Velocities (cm/s)			
	Septal TDI e′ (e′_S_)	Lateral TDI e′ (e′_L_)	Transmitral E	Transmitral A
Pre-disopyramide	4.3 ± 0.8	6.5 ± 2.0	85 ± 28	85 ± 20
Post-disopyramide	4.1 ± 1.0	6.0 ± 1.9	82 ± 23	90 ± 30
% change	−5 ± 15	−8 ± 25	−3 ± 16	+5 ± 21
p value	0.184	0.125	0.693	0.442

	Diastolic Function: Calculations		
	Septal E/e'	Lateral E/e'	E/(e′_S_+e′_L_/2)
Pre-disopyramide	21.2 ± 8.7	13.8 ± 7.9	11.7 ± 5.4
Post-disopyramide	20.7 ± 7.6	13.9 ± 9.1	11.5 ± 5.3
% change	−3 ± 22	0 ± 17	−1 ± 14
p value	0.915	0.643	0.894

	**Systolic Function**		

**LVEDV (ml)**	**LVESV (ml)**	**LVEF (%)**

Pre-disopyramide	60.5 ± 18.2	17.0 ± 6.2	72.2 ± 5.0
Post-disopyramide	63.2 ± 21.5	20.2 ± 7.3	68.0 ± 3.5
% change	+5 ± 26	+15 ± 27	−6 ± 3
p value	0.182	0.001	<0.001

Values are mean ± SD. The p values were calculated using paired Student’s *t*-test. Comparison between echocardiographic parameters obtained before disopyramide initiation (pre-disopyramide) and at the end of study (post-disopyramide).

LVEDV = left ventricular end-diastolic volume; LVEF= ejection fraction; LVESV = end-systolic volume; Lateral TDI e′(e′_L_) = early diastolic downward velocity of the lateral (free wall) mitral annulus measured at tissue Doppler; Septal TDI e′ (e′_S_) = early diastolic downward velocity of the medial (septal) mitral annulus measured at tissue Doppler; Transmitral A = late diastolic transmitral flow velocity (during atrial systole); Transmitral E = early diastolic flow velocity through the mitral valve.

### In vitro study on human myocardium

Samples from 20 patients with HCM undergoing surgical myectomy for drug-refractory symptoms were assessed. Clinical data at pre-operative assessment are shown in Supplemental Table 1.

#### Disopyramide Reduces Force and Accelerates the Kinetics of Isometric Twitches

**Figure 1:**
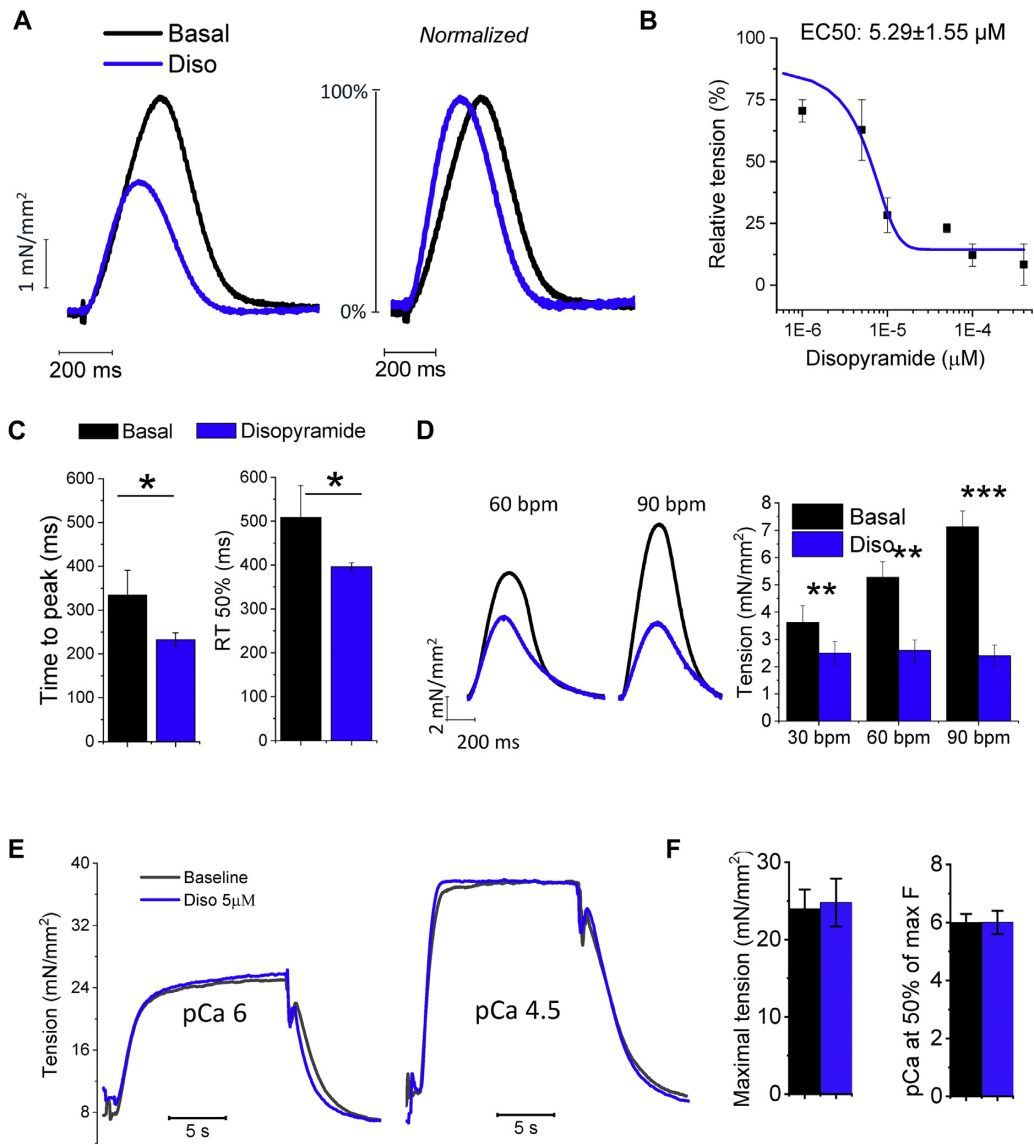
Effects of Disopyramide in Myocardial Mechanics in Intact and Skinned Trabecuale **(A)** Representative superimposed force twitches elicited at 0.5 Hz in hypertrophic cardiomyopathy (HCM) trabeculae in the absence **(black trace)** and presence **(blue trace)** of disopyramide 5 μmol/l (Diso). **(B)** Relationship between the negative inotropic effect of disopyramide and its concentration in the extracellular fluid; calculated concentration at which 50% of the maximal effect is obtained (EC50) is shown. Mean ± SEM from 5 trabeculae, 5 patients. **(C)** Time from stimulus to peak and time from peak to 50% relaxation (RT50%) of force twitches elicited at 1 Hz at baseline **(black)** and in the presence of disopyramide **(blue). (D)** Effects of disopyramide at different stimulation frequencies. Disopyramide slightly reduces the slope of force-frequency relationship at higher pacing rates. **(C,D)** Mean ± SEM from 13 trabeculae, 10 patients (ID# 1, 2, and 5 to 12). **(E)** Disopyramide in skinned trabeculae from patients with HCM. As shown in these superimposed traces from an HCM trabecula, disopyramide does not reduce isometric force at intermediate Ca activation level (pCa 6) or at maximal myofilament activation (pCa 4.5). **(F)** Maximal tension and pCa at one-half of maximal tension (Ca sensitivity) in HCM trabeculae, in the absence and presence of disopyramide. Means ± SEM from 6 trabeculae, 3 patients (ID# 12, 15, and 16). **(C,D,F)** *0.05 > p > 0.01; **0.01 > p > 0.001; ***p < 0.001; linear-mixed models. bpm = beats/min.

Disopyramide was added to standard perfusion solution while recording isometric tension from intact contracting trabeculae dissected from the endocardial surface. As expected, the drug displayed a consistent negative inotropic effect (Figure 1A). To assess the concentration dependency of this effect, we exposed the muscles to different concentrations of disopyramide (Figure 1B). Calculated disopyramide concentration at 50% of maximal effect on isometric twitch amplitude was 5.29 ± 1.55 μmol/l. We therefore decided to employ the drug at 5 μmol/l for all the following experiments; notably, 5 μmol/l corresponds to the average plasma concentration of disopyramide measured in patients under a standard treatment regimen (25). Importantly, 5 μmol/l disopyramide hastened isometric twitch kinetics in HCM trabeculae: both time to peak and relaxation time were reversibly shortened by the application of the drug (Figures 1A and 1C). We tested the effects of disopyramide at different stimulation frequencies (Figure 1D): the reduction of steady-state isometric twitch force was more pronounced at higher pacing rates as compared with lower rates. Isometric twitch force was reduced by 33 ± 5% at 0.5 Hz (30 beats/min) and by 62 ± 10% at 1.5 Hz (90 beats/min; p = 0.015 vs. 0.5 Hz; data from 13 trabeculae in 10 patients, calculated using linear-mixed models).

#### Disopyramide Has No Direct Effects on Myofilament Contraction

We tested the effects of disopyramide on demembranated trabeculae from 3 patients with HCM (Figure 1E). Disopyramide (5 μmol/l) did not affect maximal force obtained when exposing trabeculae to an activating solution with pCa 4.5 (Figure 1F). Force generation at lower [Ca^2+^], determining submaximal tension development, was also unaffected by disopyramide; we concluded that disopyramide does not modify myofilament Ca sensitivity (Figure 1F). Moreover, disopyramide did not alter the energy cost of tension generation (Supplemental Figure 1). The same results were obtained in demembranated trabeculae from healthy donors.

#### Disopyramide Reduces Diastolic Ca^2+^ and Hastens the Kinetics of Ca^2+^ Transients

**Figure 2:**
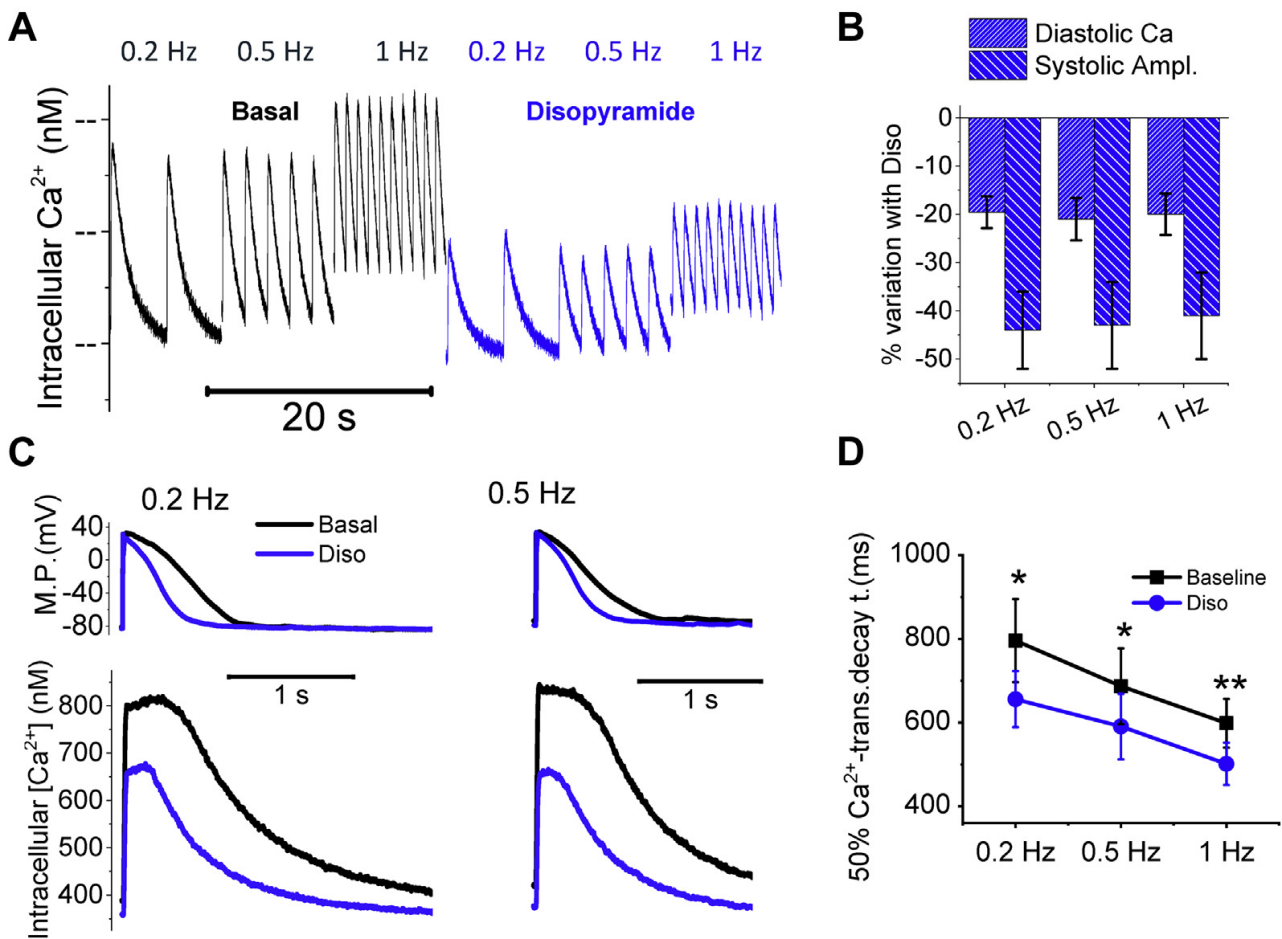
Effects of Disopyramide on Intracellular Ca^2+^ **(A)** Representative intracellular Ca^2+^ traces recorded during regular stimulation at 0.2, 0.5, and 1 Hz, in the absence **(left, black)** and presence of disopyramide 5 μmol/l (Diso) **(blue traces on the right). (B)** Percentage of variation of diastolic [Ca^2+^]_i_, and Ca-transient amplitude (Systolic Ampl) with the application of disopyramide 5 μmol/l with respect to baseline in hypertrophic cardiomyopathy cardiomyocytes, during regular stimulation at 0.2, 0.5, and 1 Hz at steady state. **(C)** Representative superimposed action potentials **(top)** and simultaneously recorded Ca transients **(bottom)** at baseline **(black traces)** and in the presence of disopyramide **(blue traces),** elicited at 0.2 Hz **(left)** and 0.5 Hz **(right). (D)** Time from peak to 50% decay of Ca transients at baseline **(black)** and in the presence of disopyramide 5 μmol/l **(blue),** elicited at 0.2, 0.5, and 1 Hz. **(B to D)** Mean ± SEM from 26 cardiomyocytes from 7 patients with hypertrophic cardiomyopathy (ID# 5 to 9 and 11 to 13). *0.05 > p > 0.01; **0.01 > p > 0.001; linear-mixed models. MP = membrane potential.

Disopyramide was tested at 5 μmol/l in patch-clamped ventricular cardiomyocytes while simultaneously recording Ca^2+^ transients and AP during stimulation at different frequencies (Figures 2A and 2C). The drug reduced the amplitude of Ca^2+^ transients at all frequencies (Figure 2B), hastened the kinetics of Ca^2+^ transient rise and decay (Figures 2C and 2D, Table 3) and reduced diastolic Ca^2+^ concentration (Figures 2A and 2B, Table 3).

**Table 3:** Effects of Disopyramide on AP and [Ca^2+^]_i_

	0.2 Hz	Baseline	Disopyramide	% Change	p Value
[Ca^2+^]_i,_ nmol/l					
	Diastolic Ca	235 ± 44	189 ± 31	−22 ± 8	0.015
	Ca^2+^-transient amplitude	336 ± 53	188 ± 34	−44 ± 15	0.006
Ca^2+^-transient kinetics, ms					
	Time to peak	151 ± 15	128 ± 12	−15 ± 8	0.024
	90% decay	1,260 ± 99	905 ± 88	−28 ± 8	0.008
Action potentials					
	MDP, mV	−80 ± 3	−80 ± 3.	0 ± 3	>0.05
	Amplitude, mV	129.1 ± 3.2	122.2 ± 2.8	−5 ± 1	0.012
	Upstroke, V/s	139 ± 18	97 ± 12	−30 ± 9	0.002
	APD20, ms	381 ± 44	284 ± 40	−25 ± 9	0.017
	APD50, ms	653 ± 63	469 ± 61	−28 ± 7	<0.001

Values are mean ± SEM. The p values were calculated using linear mixed models. Additional data from action potentials and Ca transients were recorded in hypertrophic cardiomyopathy cardiomyocytes stimulated at 0.2 Hz before and during exposure to disopyramide. Data from 28 HCM cardiomyocytes isolated from 8 hypertrophic cardiomyopathy patient samples (ID# 3 to 6, 9, and 11 to 13). Diastolic Ca is the diastolic concentration of Ca during regular stimulation. Ca^2+^-transient amplitude is the difference between peak systolic Ca and diastolic Ca. Time to peak is the measurement from stimulus to peak. The 90% decay is the time from peak to 90% decay of Ca transients. Upstroke is the upstroke speed of the action potentials.

APD20 (APD50) = action potential duration at 20% (50%) of repolarization; MDP = mean diastolic potential.

#### Disopyramide Reduces AP Duration and the Frequency of Afterdepolarizations

**Figure 3:**
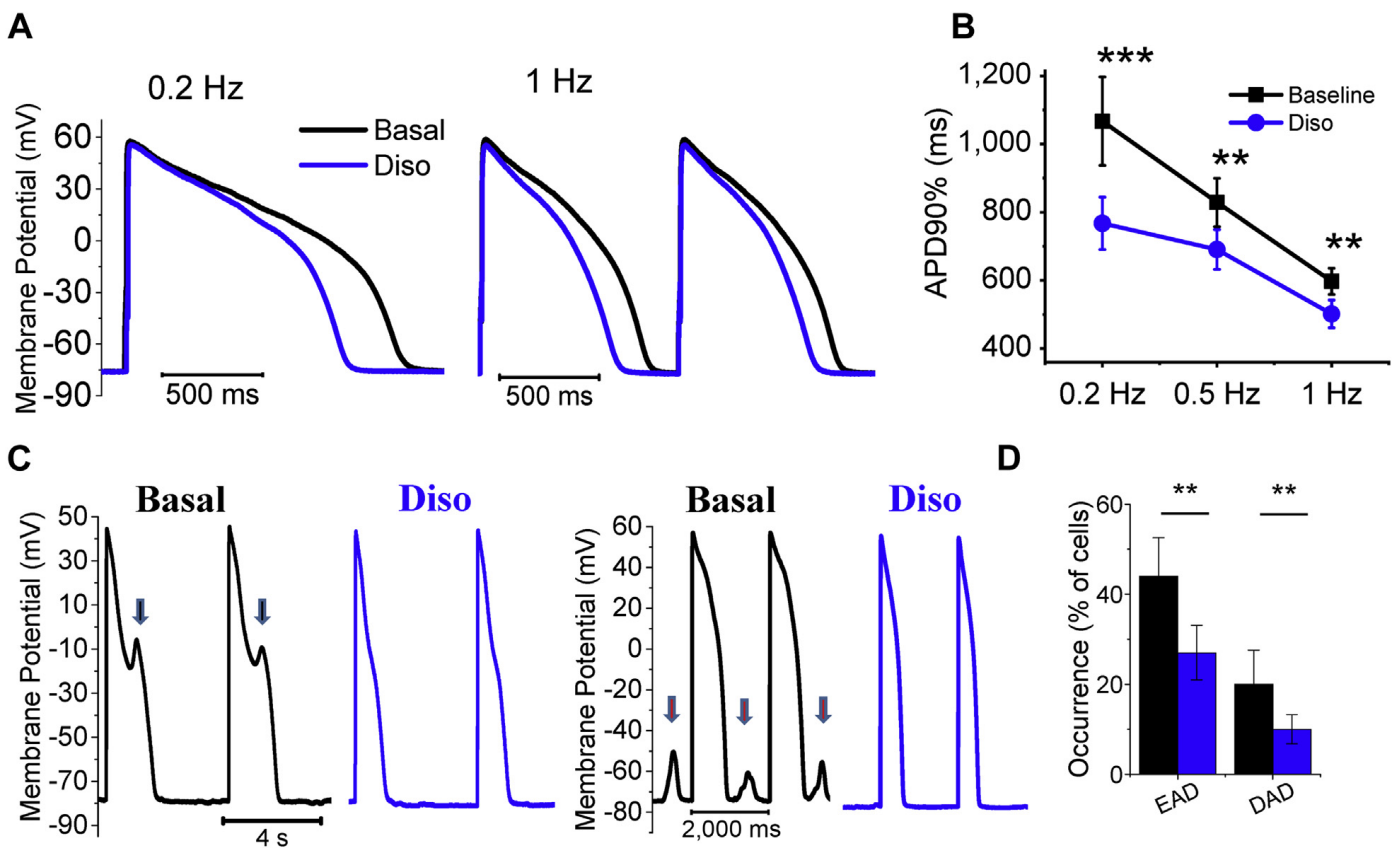
Effects of Disopyramide on AP and Cellular Arrhythmias **(A)** Representative superimposed action potentials (AP) at baseline **(black traces)** and in the presence of disopyramide 5 μmol/l (Diso) **(blue traces),** elicited at 0.2 Hz **(left)** and at 1 Hz **(right). (B)** Action potential duration at 90% of repolarization (APD90%) at baseline **(black)** and in the presence of disopyramide **(blue). (C)** Representative action traces at baseline **(black traces)** and in the presence of disopyramide 5 μmol/l **(blue traces),** elicited at 0.2 Hz pacing rate. Disopyramide suppresses early afterdepolarizations (EAD) **(arrows). (D)** Percentage of HCM cardiomyocytes showing at least 2 EAD or delayed afterdepolarizations (DAD) during 3 min of continuous stimulation, at baseline **(black)** and in the presence of disopyramide **(blue). (B,D)** Mean ± SE from 28 hypertrophic cardiomyopathy cardiomyocytes from 8 patients with hypertrophic cardiomyopathy (ID# 3 to 6, 9, and 11 to 13). **0.01 > p > 0.001; ***p < 0.001; linear-mixed models.

Disopyramide (5 μmol/l) shortened the duration of AP in HCM cardiomyocytes (Figures 2C and 3A, Table 3) at all frequencies (Figure 3B) and the effect was more pronounced at lower rates. For instance, average AP shortening at 0.1 Hz was 27 ± 5% and is comparable to that observed in human HCM cardiomyocytes with 10 μmol/l of ranolazine (17). In addition, disopyramide reduced AP amplitude and upstroke speed (Table 3). Finally, disopyramide reduced the incidence of spontaneous afterdepolarizations in HCM cardiomyocytes, both early afterdepolarizations (EAD) (occurring during the AP plateau) and delayed afterdepolarizations (DAD) (occurring during the diastolic phase), occurring during 3 min of regular stimulation (Figures 3C and 3D).

#### Disopyramide Inhibits Peak and Late Na^+^, Ca^2+^, and K^+^ Currents

**Figure 4:**
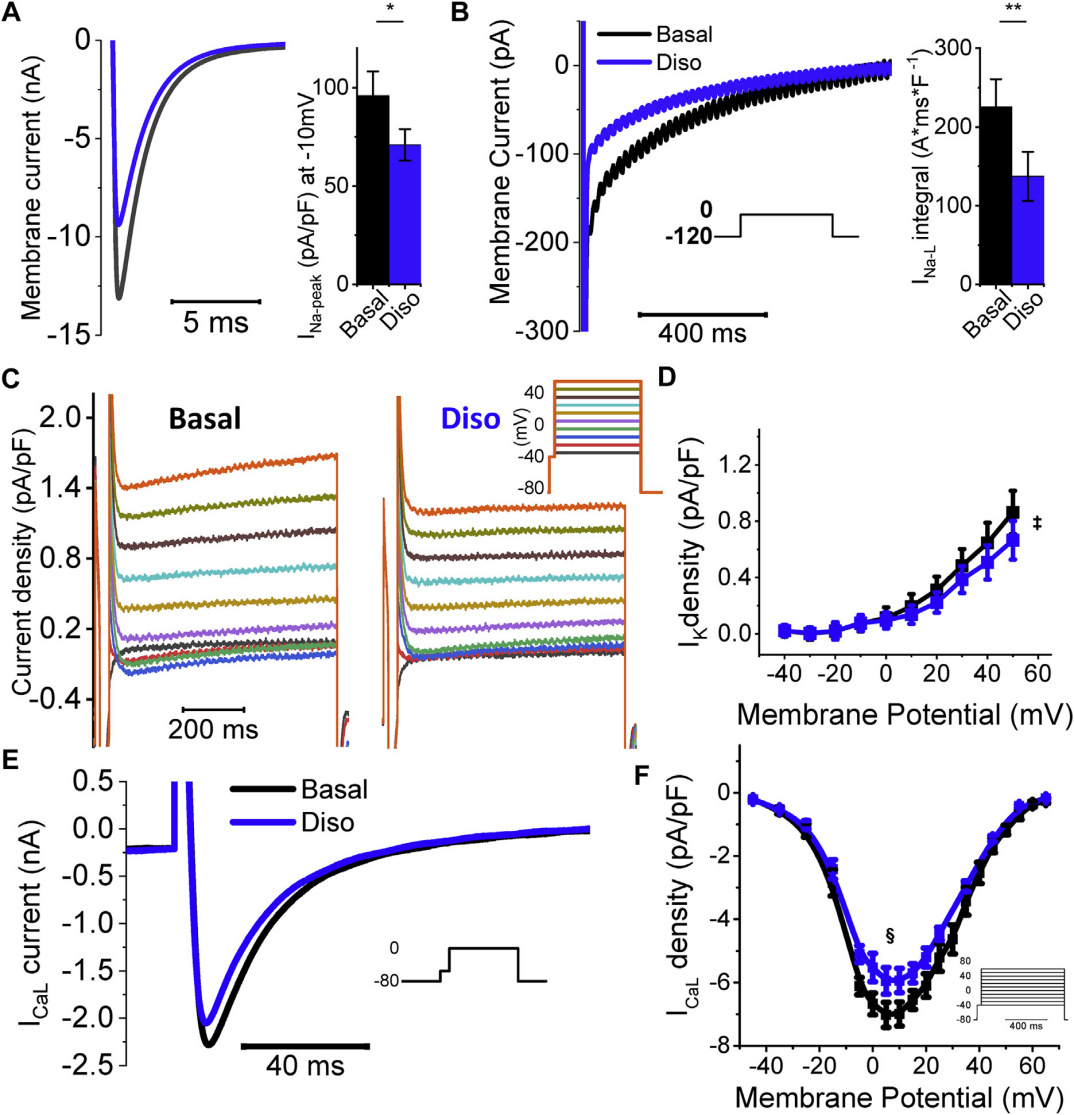
Effects of 5 μmol/l Disopyramide on Ion Channels **(A, left)** Representative peak Na current traces elicited at −10 mV from −120 mV resting potential, in a hypertrophic cardiomyopathy cardiomyocyte in the absence **(black)** and presence of disopyramide (Diso) **(blue trace). (Right)** Average peak Na^+^ current (I_Na-peak_) density at −10 mV; effect of disopyramide. **(B, left)** Representative late Na^+^ current (I_NaL_) traces elicited at -10 mV from −120 mV resting potential, in the absence and presence of disopyramide. **(Right)** Average integral of the area of the current between 50 and 750 ms after onset of the −10 mV clamp pulse, normalized by cell capacitance, calculated in the absence and presence of disopyramide in hypertrophic cardiomyopathy cardiomyocytes. **(A,B)** Mean ± SEM from 22 cardiomyocytes, 5 patients (ID# 14 to 18). *0.05 > p > 0.01; **0.01 > p > 0.001; linear-mixed models. **(C)** Delayed-rectifier K currents (I_K_) elicited at different voltages in hypertrophic cardiomyopathy cardiomyocytes (see inset for color codes), in the absence of disopyramide **(left)** and in its presence **(right). (D)** Average I_K_ current density in the absence and presence of disopyramide at different voltages. Mean ± SEM from 10 cardiomyocytes, 4 patients (ID# 14, 16, 19, and 20). ^‡^p < 0.05 for voltages ≥+20 mV, linear-mixed models used to compare each coupled pair of values. **(E)** Representative L-type Ca^2+^ current (I_Ca-L_) traces elicited at 0 mV from −80 mV resting voltage. **(F)** Average I_Ca-L_ current density in the absence and presence of disopyramide at different voltages. Mean ± SEM from 12 cardiomyocytes, 3 patients (ID# 15, 17, and 18). ^§^p < 0.05 for voltages between −10 mV and +30 mV, linear-mixed models.

We previously have shown that in HCM cardiomyocytes there is markedly enhanced I_NaL_, slightly increased I_Ca-L_, and lower I_K,_ leading to prolonged AP duration (15). We assessed Na^+^ currents in HCM cardiomyocytes during voltage clamp on depolarization to −10 mV. Peak current was measured in the first 10 ms of depolarization (Figure 4A), whereas I_NaL_ was estimated by integrating the residual inward current (50 to 800 ms after onset) (Figure 4B). In HCM myocytes, disopyramide (5 μmol/l) reduced peak Na^+^ current by 22 ± 4% and greatly decreased I_NaL_ integral by 45 ± 6% (21 myocytes, n = 5).

We then assessed the effects of disopyramide on I_K_, measured at steady state during depolarization at different potentials (Figure 4C). Disopyramide (5 μmol/l) exerted a small but significant inhibitory effect on I_K_ currents in HCM cardiomyocytes (Figure 4D). Notably, the density of steady-state I_K_ at baseline is reduced in our HCM myocytes as compared with control myocardium (Supplemental Figure 2). Moreover, disopyramide reduced the amplitude of I_Ca-L_ in HCM cardiomyocytes (Figures 4E and 4F); the average I_Ca-L_ reduction was 16 ± 4% (mean of 16 myocytes from 4 patients).

Taken together, the observed effects of disopyramide on Na^+^, K^+^, and Ca^2+^ currents explain the net reduction of AP duration by disopyramide (Figure 3). In HCM septal cells, in the presence of lower I_K_ (15), slightly increased I_Ca-L_, and markedly enhanced I_NaL_, the effect of disopyramide is a net decrease of depolarizing currents, ultimately leading to AP shortening. On the contrary, in control human ventricular myocytes, where the expression of I_K_ is preserved and I_NaL_ is small (15), inhibition of human ether-à-go-go-related gene (hERG) K^+^ current by disopyramide (26) prevails over the reduction of I_Ca-L_ and I_NaL_, ultimately leading to a slight AP prolongation (Supplemental Figure 3), in agreement with previous reports (27).

#### Disopyramide Inhibits RyR

**Figure 5:**
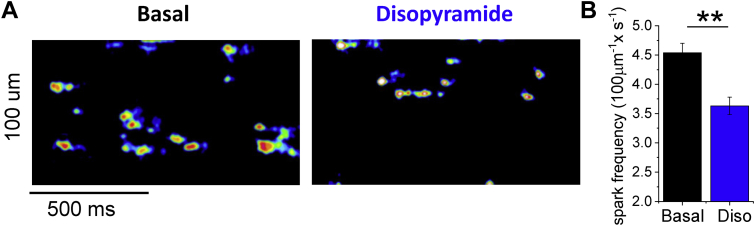
Effects of 5 μmol/l of Disopyramide on Ca Sparks in Permeabilized Myocytes **(A)** Calcium sparks in permeabilized cardiomyocytes from the left ventricle of transgenic hypertrophic cardiomyopathy mice (R92Q-TnT mutation): the representative kymographic images show confocal line scans traced along the longitudinal cell axis, displaying fluorescence signals from the Ca-selective dye, recorded at a speed of 512 lines/s while bathing the permeabilized myocytes in an intracellular solution containing 150 nmol/l [Ca^2+^] at room temperature. Spontaneous Ca sparks are visible as spontaneous elevations of local [Ca^2+^] signal lasting 20 to 40 ms. Representative traces from cells in the absence **(left)** and presence of disopyramide (Diso) **(right)** in the bathing solution. Kymographic images are reported in false color (16-colors lookup table) after thresholding and filtering (Gaussian smoothing with 2*px* sigma). **(B)** Frequency of spontaneous Ca sparks per 100 μmol/l of scanned line length, in the absence and presence of 5 μmol/l disopyramide. Mean ± SEM from 74 cardiomyocytes (basal) and 63 cardiomyocytes (disopyramide) isolated from 4 mouse hearts. **0.01 > p > 0.001, unpaired Student’s *t*-test.

Similar to other class I antiarrhythmics (28), disopyramide exerts an inhibitory effects on the ryanodine receptor (RyR). We tested the effects on RyR channels by evaluating the rate of Ca sparks in permeabilized ventricular myocytes isolated from a transgenic HCM mouse model carrying the R92Q-TnT mutation (Figure 5A). The frequency of Ca^2+^ sparks in myocytes incubated with 5 μmol/l of disopyramide was lower than that observed in vehicle-treated cells (Figure 5B).

### Disopyramide decreases ventricular dispersion of repolarization in silico

**Figure 6:**
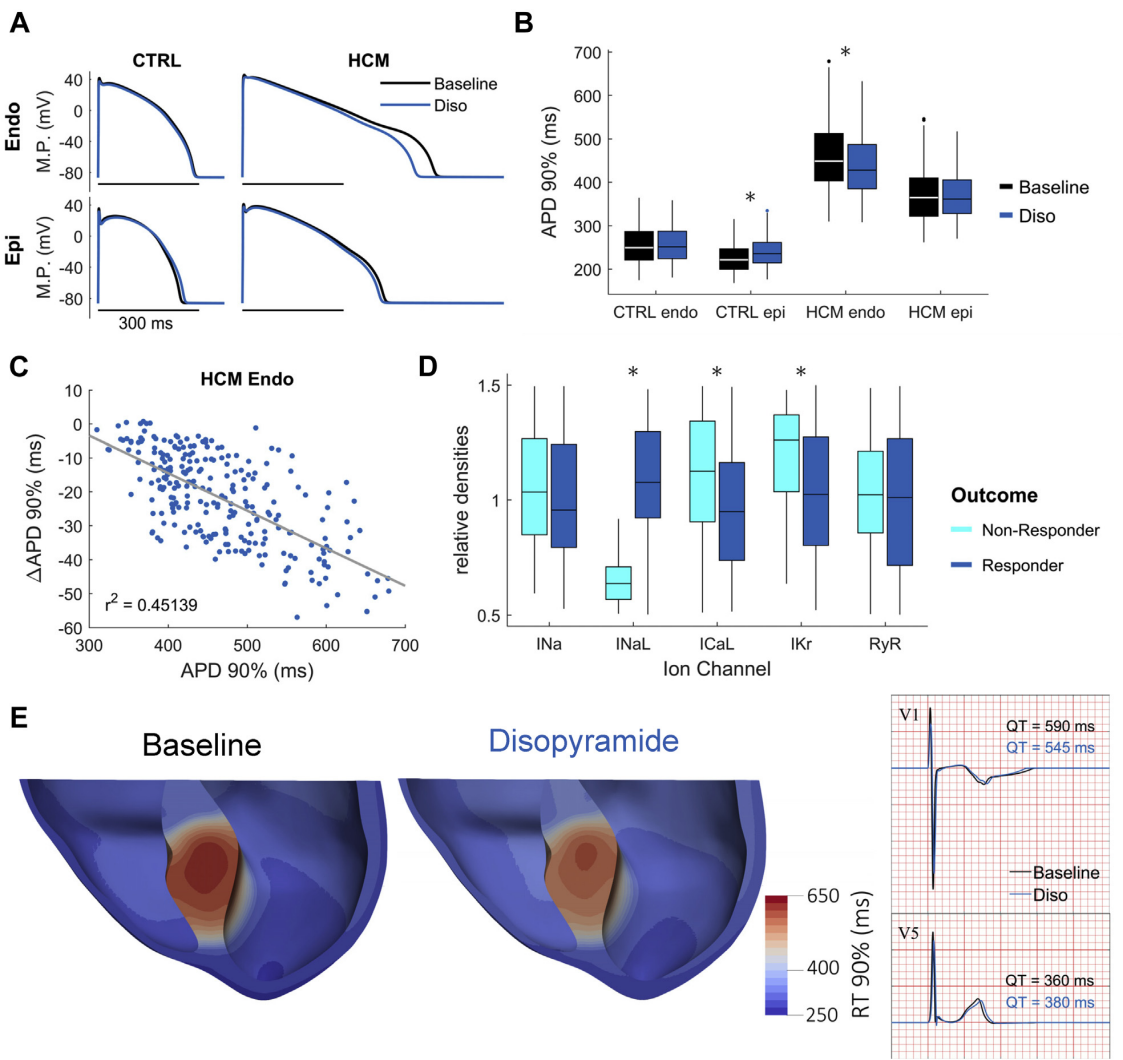
Modeling Results on the Action of Disopyramide on the Dispersion of Repolarization **(A)** Representative superimposed action potentials at baseline **(black traces)** and in the presence of disopyramide 5 μmol/l **(blue traces)** in endocardial (Endo) and epicardial (Epi) models of human ventricular cardiomyocytes at 1 Hz pacing. **(B)** APD90% at baseline **(black)** and in the presence of disopyramide 5 μmol/l **(blue),** for the different cell types. *p < 0.05, paired Student’s *t*-test. **(C)** Endocardial APD shortening in HCM endocardial cardiomyocytes under disopyramide 5 μmol/l action as a function of baseline APD90%. **(D)** Cellular mechanisms underlying endocardial APD shortening in responders **(dark blue)** versus nonresponders (**light blue)** to disopyramide. *p < 0.05, unpaired Student’s *t*-test. **(E)** RT90% in a ventricular model of obstructive HCM. Disopyramide reduces maximum dispersion of repolarization in the septal hypertrophic region while only slightly prolonging repolarization in nonhypertrophic epicardium, leading to QT shortening in septal compared with modest QT prolongation in lateral precordial electrocardiogram leads (V_1_ and V_5_ shown). CTRL = control model; I_Kr_ = rapid delayed-rectifier K^+^ current; RyR = ryanodine receptor; other abbreviations as in Figures 1, 2, 3, and 4.

The effects of 5 μmol/l of disopyramide were modeled in silico based on the above-mentioned characterization of drug action. In agreement with the experimental findings, 5 μmol/l of disopyramide yielded AP shortening in HCM endocardial cardiomyocytes and left AP duration unaltered in control endocardium (Figures 6A and 6B). Due to transmural differences in ion channel expression, this trend was reversed in epicardial cardiomyocytes, where disopyramide slightly prolonged AP duration in control cells (Figures 6A and 6B). The magnitude of AP duration at 90% of repolarization shortening in HCM endocardial cells was inversely correlated with basal AP duration at 90% of repolarization (Figure 6C). Further analysis of responders to drug action indicates that I_NaL_ density is the primary determinant of the extent of AP shortening due to disopyramide (Figure 6D).

In the reconstructed ventricles of a patient with obstructive HCM, 5 μmol/l disopyramide reduced conduction velocity, increasing total activation time by 11 ms (Supplemental Figure 4), comparable to clinical data (QRS prolongation at ECG, see Table 1). In the region of septal hypertrophy, 5 μmol/l of disopyramide markedly decreased dispersion of repolarization times (baseline: 529 ± 62 ms, 625 ms maximum; 5 μmol/l of disopyramide: 504 ± 50 ms, 585 ms maximum) and slightly prolonged repolarization in the nonhypertrophic epicardium (Figure 6E). This heterogeneous ventricular action resulted in QT shortening in septal precordial ECG leads along with modest QT prolongation in the lateral ones (Figure 6E), hence explaining the apparent discrepancy between AP shortening and the clinical QTc prolongation observed in patients with HCM following disopyramide intake.

## Discussion

In the present work, we have investigated the effects of disopyramide in patients with obstructive HCM and on isolated HCM cardiomyocytes harvested from patients undergoing surgical septal myectomy. The results shed light on the agent’s biophysical mechanisms of benefit, and its demonstrated clinical safety in patients with HCM. In addition, the suppressive effect of disopyramide on afterdepolarizations in vitro raises the possibility that disopyramide may reduce ventricular arrhythmia propensity in vulnerable patients with HCM.

### Negative inotropic effect in obstructive patients

In patients with obstructive HCM, the beneficial effect of disopyramide is that it decreases LV contractility, specifically by decreasing early LV ejection flow acceleration (29). In patients with obstructive HCM, the mitral valve apposes with the septum very early in systole, and the duration of mitral-septal contact correlates with gradient magnitude, because of the amplifying feedback loop in which the presence of a gradient pushes the valve further into the septum. Thus, to reduce obstruction-related gradients, the impact of any negative inotropic medication must be very early in systole, before the onset mitral-septal contact. By decreasing early systolic ejection acceleration, disopyramide decreases early drag forces on the mitral valve, delaying or abolishing mitral-septal contact. Systolic anterior motion results from an equilibrium between the displacing anteriorly directed force of ejection flow striking the mitral valve, versus restraining forces, from the papillary muscles and chordae (30). Disopyramide displaces this equilibrium toward restraint by dint of its effects on early flow acceleration. Interestingly, despite the decreased velocity of pressure generation (dP/dt) during early systole and the lower peak LV pressure, disopyramide results in a modest reduction of global systolic function (5% to 6% decrease of ejection fraction in our study, in line with previous observations). In normal subjects, disopyramide causes a prolongation of total ejection time (1); in subjects with HCM, however, where ejection time is prolonged due to obstruction, the relief of obstruction by disopyramide leads to a 12% shorter ejection time (31). Moreover, disopyramide has a more potent gradient-lowering effect than other negative inotropic agents given for obstructive HCM (beta blockers and verapamil). In a head-to-head comparison in individual patients with HCM by intravenous administration of the 3 agents on sequential days, investigators found a 59% reduction of gradient with disopyramide, a 19% reduction with propranolol, and only 8% reduction with verapamil 11, 32. On average, 60% to 70% of patients started on disopyramide will experience a significant drop in LVOT gradient and improvement in symptoms 5, 6. Whereas the gradient-lowering effect of disopyramide shows a clear dependency on dose and plasma levels 1, 3, 33, the negative inotropic effect is always very modest and does not vary within the clinically relevant range of doses (up to 300 mg twice daily). These observations suggest that symptomatic gradients can be effectively lowered by negative inotropic agents without largely reducing ejection fraction. This may be related to the fact that most patients with obstructive HCM are pathologically hyperkinetic. In HCM hearts, negative inotropic drugs may counteract LV hypercontractility and reduce early ejection acceleration while at the same time allowing a more efficient LV emptying due to loss of obstruction. Thus, the impact of negative inotropic drugs on end-systolic volume will be very limited, explaining the modest reduction of ejection fraction.

In the current study, disopyramide lowered resting LVOT gradients from mean 58 to 25 mm Hg after 3 months. This was similar to a prior report in 221 patients using disopyramide with an average dose of 500 mg/day, where resting LVOT gradients decreased from 63 to 25 mm Hg after 4.5 years (6). Symptoms and gradient in 64% of patients of this cohort could be managed pharmacologically, while 36% ultimately underwent septal reduction.

### Cellular mechanisms underlying the negative inotropic action of disopyramide

Our in vitro force assessments using human HCM samples confirm the negative inotropic action of disopyramide observed in patients. The results of our studies show that this effect is the result of the inhibitory action of disopyramide on multiple ion channels within the cardiomyocyte.

#### Disopyramide Has No Direct Effects on Myofilaments

Disopyramide does not modify the maximal force generated by the contractile apparatus in HCM and control trabeculae, and it does not change myofilament sensitivity to Ca^2+^, suggesting that the drug does not directly target contractile proteins (Figure 6). Therefore, disopyramide must reduce force through modifications of the cardiomyocyte excitation-contraction coupling, that is, intracellular Ca^2+^ handling. By contrast, mavacamten (MYK-461) produces a negative inotropic effect by virtue of direct inhibitor of cardiac myosin (34) with no apparent off-target side effects. Mavacamten was recently tested in a small number of patients with obstructive HCM in a phase II study, showing that a clinically meaningful reduction of post-exercise LVOT gradients can be achieved with relatively small doses of the drug, while leading to a modest reduction of LV ejection fraction, on the order of 6% to 10% on average. This is in line with the idea that a large reduction of LVOT gradients in patients with obstructive HCM can be achieved using negative inotropic drugs with a minimal loss of global LV function.

#### Disopyramide Reduces the Amplitude of Calcium Transients

The negative inotropic effect of disopyramide is a consequence of the reduction of systolic intracellular Ca^2+^ levels in the cardiomyocyte, thus determining lower Ca-mediated activation of myofilaments and, ultimately, lower force. We found a number of concurrent mechanisms contributing to the reduction of Ca^2+^-transient amplitude.

#### Disopyramide Reduces Peak Na^+^ Current and I_NaL_

Early studies demonstrated that Na^+^ channel block (e.g., by tetrodotoxin) has a slight negative inotropic action per se in the myocardium. Interestingly, these studies compared the negative inotropic potency of different drugs with their efficacy in slowing down AP upstroke (a direct consequence of peak Na^+^-current inhibition) 35, 36. The ratio of negative inotropic efficacy versus Na^+^-channel blocking potency was the lowest for tetrodotoxin (0.23) and the highest for disopyramide (2.2). Disopyramide has the most negative inotropic effect of all Class I antiarrhythmics, higher than mexiletine, procainamide, and quinidine (35). As tetrodotoxin is a pure Na^+^-channel blocker, these results suggest that Na^+^-channel inhibition only slightly contributes to the negative inotropic action of disopyramide, and that disopyramide has additional Na^+^-channel–independent mechanisms that make it the most potent negative inotropic agent among class I antiarrhythmics (35).

Nonetheless, Na^+^-channel inhibition may independently contribute to the reduction of Ca^2+^-transient amplitude by disopyramide through modification of the activity of the NCX (37). When membrane potential is positive (peak and plateau of the AP) and subsarcolemmal [Na^+^] is high (due to large Na^+^ influx by peak I_Na_), NCX works in reverse mode, in other words, letting Ca^2+^ enter the cell in exchange for Na^+^ (38). The contribution of reverse-mode NCX to Ca^2+^ transients is particularly high in HCM cardiomyocytes (16). In the presence of peak and late I_Na_ inhibition by disopyramide, maximal AP voltage is reduced (Table 2) and intracellular [Na^+^] is diminished (16): these changes lead to a decrease of reverse-mode NCX activity, thus reducing Ca^2+^ entry (39). Moreover, reduction of diastolic [Na^+^] may enhance the diastolic extrusion of Ca^2+^ via the NCX (forward mode) 15, 18. The 2 mechanisms (reduced reverse- and enhanced forward-mode NCX) may contribute to reduce total Ca^2+^ load of the SR (15) and thus Ca^2+^-transient amplitude. Selective inhibition of I_NaL_ without block of peak I_Na_ (e.g., with ranolazine or eleclazine) is not sufficient to induce a negative inotropic effect under basal conditions 15, 16. Therefore, we can conclude that simultaneous inhibition of peak and late I_Na_ is required for the negative inotropic action of disopyramide.

#### Disopyramide Reduces I_Ca-L_

Disopyramide exerts a slight but relevant inhibitory effect on I_Ca-L_, which is substantially lower than that of verapamil. Nonetheless, the observed 15% inhibition of peak I_Ca-L_ by disopyramide contributed to reduce Ca^2+^-transient amplitude in ventricular myocytes, the effect being larger at higher stimulation rates. A similar rate-dependency of the negative inotropic effect is typical of pure Ca^2+^-channel blockers such as verapamil (40). At variance with verapamil (41), however, disopyramide has minimal effects on heart rate and atrioventricular conduction (Table 3), and thus can be safely combined with -beta blockers.

#### Disopyramide Inhibits RyR

Na^+^ channel blockers flecainide and propafenone stabilize the closed state of cardiac RyR channels (42). We observed that disopyramide reduced the rate of spontaneous Ca^2+^ sparks in permeabilized myocytes, in the presence of fixed intracellular [Ca^2+^]: the reduction in the spontaneous RyR opening rate is likely due to a direct effect of disopyramide on RyR channels. Notably, selective inhibition of RyR channels using JTV-519 in human ventricular trabeculae led a slight negative inotropic effect (43). Similarly, inhibition of RyR-mediated systolic Ca^2+^ release by disopyramide may contribute to the reduction of Ca^2+^-transient amplitude.

### Disopyramide shortens repolarization in HCM cardiomyocytes: Molecular mechanisms

Early studies in healthy myocardium showed that disopyramide prolongs ventricular AP (13) due to decreased I_Kr_ current by direct inhibition of hERG channels (44). Similarly, we observed a slight AP prolongation in cardiomyocytes from patients without HCM (Supplemental Figure 3), also confirmed by modeling studies (Figure 6A). Interestingly, the AP prolonging effect in control cardiomyocytes was shown to be prevalent at concentrations below 8 μmol/l, whereas at higher concentrations, disopyramide tended to shorten AP 13, 45. At low concentrations, hERG inhibition prevails over I_NaL_ and I_Ca-L_ block, leading to a net decrease of repolarizing currents and AP prolongation; at higher concentrations, however, after maximal hERG inhibition is reached, the inhibition of depolarizing Ca^2+^ and Na^+^ currents becomes prevalent, leading to AP shortening in healthy myocardium. Based on our observations, we can speculate that the latter behavior occurs in HCM cardiomyocytes even at low, clinically relevant concentrations (such as 5 μmol/l, which we used in this work), due to the decreased expression of hERG channels in HCM myocardium (15). Moreover, I_NaL_ and I_Ca-L_ are both increased in HCM versus control myocardia (15); therefore, their inhibition by disopyramide leads to greater AP shortening, especially at low pacing rates. Simulation studies suggested that AP shortening by disopyramide is more pronounced in cells that have larger I_NaL_ and smaller I_Kr_ (Figures 6C and 6D). Taken together, the altered balance of depolarizing and repolarizing currents in cardiomyocytes from the hypertrophic septum of patients with HCM causes disopyramide to decrease net depolarizing currents (Na^+^ and Ca^2+^) without substantial reduction in repolarizing K^+^ currents, ultimately shortening AP plateau duration (Figure 2, Table 1).

### Shortening of AP duration and QTc prolongation: An apparent contrast

In the 39 patients begun on disopyramide, QTc interval increased from 458 ± 22 to 486 ± 27, an average 5.8% rise after 3 months. The shortening of AP observed in HCM cardiomyocytes in vitro is in apparent contrast with the slight QTc prolongation in patients. However, prolongation of QTc interval with disopyramide depends, at least in part, on the prolongation of QRS interval, reflecting delayed intraventricular electrical conduction velocity due to peak Na^+^-current inhibition (Table 1) (46). However, even if we consider only the repolarization phase (JT interval), disopyramide still has a significant prolonging effect. All the cardiomyocytes analyzed in this work were isolated from the subendocardial region of the basal septum, the most hypertrophied region in patients with obstructive HCM. If the degree of cardiomyocyte electrical changes is heterogeneous across the LV and mirrors the asymmetrical distribution of hypertrophy, cardiomyocytes from other regions of the LV are likely to be similar to control cardiomyocytes. Therefore, we constructed a 3-dimensional model of the whole LV from an obstructive patient (Figure 6E) where we assumed that the HCM-specific ion current abnormalities we observed in isolated cardiomyocytes from septal myectomy samples 15, 23 were limited to the hypertrophied septal region. In agreement with this assumption, the spatial dispersion of QTc interval is greatly increased in patients with HCM with asymmetrical hypertrophy (47). In this model, we found that disopyramide shortens the duration of septal AP while slightly prolonging the AP of the remaining LV myocardium, ultimately resulting in a slight prolongation of the global QT interval in the reconstructed ECG trace. In line with that prediction, the average prolongation of QTc interval in patients with HCM with disopyramide (5% to 6%) is lower than the average QT prolongation in healthy patients taking a similar dose of disopyramide (10% to 15%) 1, 48. The model predicts that disopyramide determines marked reduction of QT dispersion across the different LV regions, as well as transmurally. Because the magnitude of the AP-shortening effect of disopyramide is linearly related with the AP duration at baseline (Figure 6C), the effect would be greater in cells with longer AP, globally reducing the heterogeneity of AP duration among different LV regions. In line with that, we observed in patients that the QTc prolonging effect of disopyramide is inversely related to the baseline QTc interval: patients with an initially longer QTc interval (>457 ms) had a smaller increase in QTc interval after disopyramide than did patients with shorter initial QTc interval. Interestingly, spatial QT dispersion is a substrate for sustained re-entrant arrhythmias and is related with the occurrence of nonsustained ventricular tachycardia in patients with HCM (47); therefore, its reduction by disopyramide could be antiarrhythmic.

### Effect on early and late afterdepolarizations: Antiarrhythmic potential

We observed a reduction of the cellular triggers of arrhythmias in HCM cardiomyocytes treated with disopyramide, that is, EAD and DAD (Figure 3). The risk of EAD is directly associated with AP prolongation (49). The shortening of AP by disopyramide is therefore the main mechanism behind the reduction of EAD (15). DAD result from the activation of the electrogenic (depolarizing) NCX secondary to spontaneous diastolic Ca^2+^ release from the SR (50). The likelihood of DAD is therefore increased in all conditions leading to increased open probability of RyR during diastole, that is increased SR Ca^2+^ load, increased diastolic cytosolic [Ca^2+^] or altered intrinsic properties of the RyR (e.g., phosphorylation by Ca^2+^/calmodulin-dependent protein kinase II). All these features are present in HCM cardiomyocytes, explaining the observed increase of DAD with respect to control cells (15). Interestingly, we observed that disopyramide reduced diastolic cytosolic [Ca^2+^] (Figure 2) and SR Ca^2+^ content and stabilized the closed state of RyR (Figure 5), thus lowering the likelihood of diastolic Ca^2+^ waves.

### Safety of disopyramide in clinical practice

**Figure 7:**
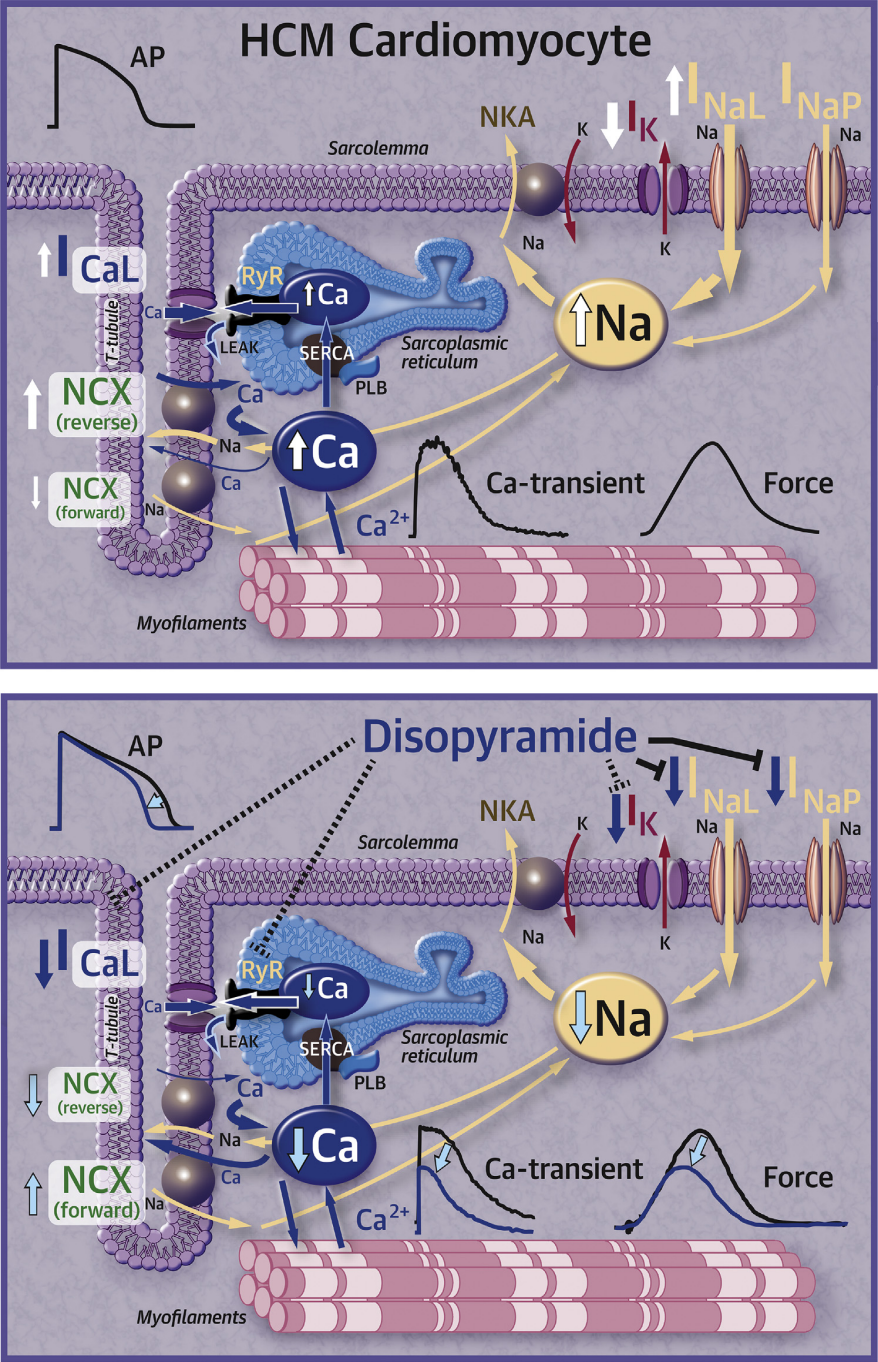
Effects of Disopyramide in HCM Cardiomyocytes **(Top)** In HCM cardiomyocytes, I_Ca-L_ and I_NaL_ are increased, while I_K_ is markedly decreased, leading to prolonged APs; Na overload impairs NCX, contributing to cytosolic Ca-overload. **(Bottom)** Disopyramide inhibits I_Na-peak_ (I_NaP_), I_NaL_, I_Ca-L_ and I_K_, while also stabilizing ryanodine receptors. These effects lead to shortening of APs. Moreover, normalization of NCX function and I_Ca-L_ inhibition and RyR stabilization contribute to reduce diastolic Ca and systolic Ca-release, determining negative inotropic effects. APs = action potentials; HCM = hypertrophic cardiomyopathy; I_Ca-L_ = L-type Ca current; I_K_ = delayed-rectifier K current; I_NaL_ = Late Na current; NCX = Na^+^/Ca^2+^ exchanger; RyR = ryanodine receptor.

The multichannel inhibitory effects and the membrane stabilizing actions of disopyramide in vitro provide an explanation to the clinical observation that, even though the drug prolongs QT intervals, it does not increase arrhythmic propensity in HCM. In a multicenter study of disopyramide, with a relatively low dosage, there was a clear trend toward lower sudden cardiac death mortality in disopyramide-treated patients with HCM (5). Subsequent studies have shown that the incidence of sudden cardiac death, appropriate ICD discharges or resuscitated cardiac arrest in a cohort of over 200 patients treated with disopyramide is only 0.3% per year, as evaluated after an average follow-up of 5 years 4, 6. This rate of lethal or potentially lethal events compares favorably with the natural history of HCM and even with the long-term outcome of patients with obstructive HCM treated with surgical myectomy (51). Disopyramide appears to have an intrinsic safety mechanism in HCM cells, whereby the QT prolonging effects are most evident in cells with short baseline AP duration and are overcome by QT shortening effects in those with longer AP. Combined with its potential protective action from ventricular arrhythmias, this provides disopyramide with a very favorable profile for clinical use in HCM. Following the results of this work, an investigation with prolonged arrhythmia event monitoring before and after disopyramide would be of great interest. In addition to its beneficial electrophysiological effects described herein, the hemodynamic effect of gradient reduction is likely to be antiarrhythmic by decreasing myocardial work and supply-demand ischemia, and by improving myocardial energy efficiency. Despite the demonstrated electrophysiologic and clinical safety, we continue to believe it is prudent to avoid concomitant administration of other QT-prolonging medications along with disopyramide (Figure 7).

### Study limitations

First, in this work, we only studied cardiomyocytes isolated from the hypertrophied upper septum; therefore, we could not verify whether the effects of disopyramide are different in cells from other less affected regions. Second, disopyramide hastens the decay of Ca^2+^ transients and accelerates myocardial relaxation in HCM myocardium (Figures 1 and 2), as a consequence of the increased Ca^2+^-extrusion activity of NCX 15, 18 (Figure 5). Such effect is expected to improve diastolic function in vivo. However, echocardiographic data from our patients did not show any amelioration of active mechanical diastolic parameters (Table 4). This is in line with previous results obtained in patients with HCM using ranolazine, which failed to improve diastolic parameters, despite reducing circulating pro–B-type natriuretic peptide (52). Third, we did not directly evaluate the effects of disopyramide on atrial or ventricular arrhythmia burden in the patients included in the clinical study, as no ambulatory ECG monitoring was performed. Fourth, our results do not provide clear mechanistic insights into why up to 30% of patients with obstructive HCM started on disopyramide are nonresponders. Given the relatively small number of patient samples, we could not observe a subset of them showing minimal or no response to the drug. However, as the response to disopyramide appears to be higher in cells with more pronounced electrophysiological changes (e.g., increase of I_NaL_ and decrease of I_K_ [see Figures 6C and 6D]) and the severity of electrical remodeling varies substantially among different patients (as we previously reported in [15]), interpatient variability in the severity of cellular abnormalities may underlie the differences in the clinical response to disopyramide. Moreover, prior clinical investigations have shown that a subset of patients with a combination of both high resting gradients >85 mm Hg and long anterior mitral leaflets ≥33 mm Hg have a suboptimal response to oral disopyramide due to a combination of adverse anatomy and excessive leaflet slack (6). Fifth, due to the limited number of patient samples and incomplete genetic data (Supplemental Table 1), we were unable to correlate specific parameters of drug effectiveness with the different disease-causing mutated genes. As disopyramide does not directly interacts with sarcomeres (Figure 1), we believe it is unlikely that mutations in different sarcomeric genes affect the efficacy of the drug. Moreover, we previously observed that the degree of electrical abnormalities at cardiomyocyte level is similar in samples from patients carrying mutations in *MYH7* and in *MYBPC3* and patients with no sarcomeric gene mutations. As disopyramide targets ion channels, these results support the idea that the effects of the drug do not vary depending on the different causing mutations. Sixth, one-half of the patients with HCM included in this study were under disopyramide therapy prior to surgery (Supplemental Table 1); as it is unknown whether long-term disopyramide treatment alters the expression or function of ion channels and other functional cardiomyocyte proteins, we cannot exclude that this may have influenced the results of this study.

## Conclusions

Disopyramide emerges as a safe drug due to its multichannel blocking effects. Disopyramide is an effective negative inotrope that avoids interference with sarcomere protein function. This promises to enrich the pharmacological armamentarium to control obstruction as well as arrhythmias. Indeed, our data suggest a potential protective effect of disopyramide from ventricular arrhythmias mediated by suppression of afterdepolarizations and transmural re-entry; future studies aimed at evaluating the antiarrhythmic potential of disopyramide in patients with HCM are warranted. The results of this study provide a measure of reassurance to clinicians still concerned with the use of disopyramide in a complex structural disease such as HCM.

Perspectives **COMPETENCY IN MEDICAL KNOWLEDGE 1:** The effects of disopyramide in the hearts of patients with HCM are mediated by the combined inhibition of peak Na^+^ current, I_NaL_, Ca^2+^ channels, K^+^ channels, and RyR. Such multichannel inhibition explains the marked negative inotropic effect of the drug, which is responsible for the reduction of LV outflow gradients in patients with obstructive HCM. Moreover, these combined actions also result in shortening of the HCM cardiomyocyte AP, improvement of intracellular Ca overload, reduction of afterdepolarizations, and smaller dispersion of repolarization, conferring additional antiarrhythmic potential.

**COMPETENCY IN MEDICAL KNOWLEDGE 2:** Our translational study provides the mechanistic explanation for the safety and efficacy of disopyramide in patients with obstructive HCM, which has been confirmed by over 40 years of clinical use. These results support the idea that disopyramide treatment can be safely initiated in the outpatient setting and requires only an initial evaluation of the changes in QTc interval, which are usually rather small and are not associated with an increased risk of arrhythmias.

**TRANSLATIONAL OUTLOOK 1:** The negative inotropic effect of disopyramide does not depend on direct inhibition of myofilament contraction. As such, it does not appear incompatible with the new class of allosteric myosin modulators currently under investigation (mavacamten). As disopyramide and mavacamten act on different targets, they could potentially be combined with synergic effects, if the efficacy and safety of mavacamten on long-term administration is confirmed in the ongoing phase III studies.

**TRANSLATIONAL OUTLOOK 2:** The use of drugs that inhibit hERG channels and cause drug-related QT prolongation are commonly considered to be more dangerous in patients with pre-existing structural heart disease, such as HCM. This assumption does not take into consideration the disease-related changes in cardiomyocyte ion channel expression. In HCM, the baseline expression of hERG channels is reduced, so the QT prolonging effect of disopyramide (and probably that of other hERG blocking drugs) is reduced. Paradoxically, the proarrhythmic consequences of hERG-blocking QT-prolonging drugs might be less severe in hearts with structural disease and profound electrical remodeling, as compared with healthy hearts.

**TRANSLATIONAL OUTLOOK 3:** The promising antiarrhythmic properties of disopyramide in vitro suggest a role for clinical use also in patients without obstructive HCM, such as, as a less toxic alternative to amiodarone for control of atrial fibrillation.
