# Supplementary material for: Electrophysiological and Contractile Effects of Disopyramide in Patients With Obstructive Hypertrophic Cardiomyopathy: A Translational Study
Source: JACC Basic Transl Sci. 2019 Oct 9;4(7):795–813. doi: 10.1016/j.jacbts.2019.06.004 (PMC6978554; doi:10.1016/j.jacbts.2019.06.004)
Supplement: Supplemental Data [file mmc1.pdf]

ONLINE SUPPLEMENTARY CONTENTS

Translational Study of the Electrophysiological and Contractile Effects of Disopyramide in Patients with Obstructive Hypertrophic Cardiomyopathy

Raffaele Coppini, MD, PhD; Cecilia Ferrantini, MD, PhD[2]; Josè Manuel Pioner, PhD; Lorenzo Santini, MS; Zhinuo J. Wang, PhD; Chiara Palandri, MS; Marina Scardigli, PhD; Leonardo Sacconi, PhD; Pierluigi Stefàno, MD; Laura Flink, MD; Katherine Riedy, MD; Francesco Saverio Pavone, PhD; Elisabetta Cerbai, PhD; Corrado Poggesi, MD; Alessandro Mugelli, MD; Alfonso Bueno-Orovio, PhD; Iacopo Olivotto, MD and Mark V. Sherrid, MD

Index:

Supplemental Methods……………………………………………………2

Supplementary references……………………………………………..10

Supplementary Table 1……………………………………………...13

Supplementary Table 2……………………………………………...14

Supplementary Fig. 1……………………………………………......15

Supplementary Fig. 2……………………………………………......16

Supplementary Fig. 3……………………………………………......17

Supplementary Fig. 4……………………………………………......18

SUPPLEMENTAL METHODS

CLINICAL STUDY

Prospective observational study of patients with obstructive HCM treated with disopyramide: From October 2015 to January 2018, 39 patients with limiting heart failure symptoms and elevated LVOT gradients resistant to beta blockade or verapamil were initiated on Diso at NYU Langone Medical Center as previously described[1-4]. Patient gave written informed consent for the use of their clinical information. Prior to mid-2017 patients were admitted to the hospital for three days for Diso initiation[5]. After the publication of Adler et al in 2017[4], demonstrating the safety of outpatient initiation of Diso, patients were started in this fashion, albeit with outpatient ECG surveillance at 3 days, 3 weeks and 3 months. Initial Diso dosage was 250 mg twice a day, given as the control release preparation. Drug titration was performed as previously described, depending on symptomatic response, up to a maximum dosage of 300 mg twice a day. Patients were maintained on their usual beta blocker dosage, titrated to achieve a resting heart rate target of 55-60 bpm. If patients experienced vagolytic side effects (dry mouth) they were prescribed pyridostigmine sustained release to be taken as needed[3, 5]. In 9 patients (23%), Diso was ineffective or there were limiting vagolytic side effects that led to termination of drug before 3 months. In these patients, the ECGs and echocardiograms were acquired before drug termination and represented the final data point. Clinical data are expressed as means ± SD (Standard Deviation).

Echocardiography: Continuous wave Doppler was used to calculate resting LVOT gradients. Transmitral flow velocities were acquired with pulsed Doppler echocardiography at the tips of the mitral leaflets. Tissue Doppler velocities were measured at the medial and lateral mitral annulus.

IN VITRO STUDIES

Patients for cells/trabeculae studies: In vitro studies were performed at the University of Florence. Protocols were approved by the ethical committee of Careggi University-Hospital (2006/0024713; renewed May 2009). We enrolled 20 HCM patients regularly followed by our Cardiomyopathy Unit and consecutively referred to surgical myectomy for relief of drug-refractory symptoms related to LVOT obstruction. Among the 20 patients, 12 agreed to undergo mutational screening in sarcomeric genes. Clinical and genetic data are found in Supplementary Table 1.

The control cohort comprised 4 patients aged <65 years undergoing heart surgery for aortic stenosis or regurgitation and who required a septal myectomy operation due to the presence of a

bulging septum causing symptomatic obstruction. All control patients had septal thickness <14mm and preserved left-ventricular systolic function (ejection fraction >55%). Clinical data are found in Supplementary Table 2.

Tissue processing: Septal specimens were prepared as we previously described[6]. Briefly, septal samples from HCM and control patients were rapidly washed in ice-cold cardioplegic solution containing (in mmol/L): KH_2PO_4 50, $MgSO_4$ 8, HEPES 10, adenosine 5, glucose 140, mannitol 100, taurine 10 (pH 7.4 with KOH). Within 15 minutes from excision, a small portion of the tissue was frozen in liquid nitrogen and used for protein and mRNA isolation. The remaining fresh tissue is kept in ice-cold cardioplegic solution and used to isolate multicellular preparations and single cardiomyocyes. Endocardial trabeculae suitable for mechanical measurements (300-800 µm diameter) were dissected, while the remaining tissue was minced to small pieces (~1mm^3) and subjected to enzymatic and mechanical dissociation to obtain viable single myocytes, as described before[7]. In brief, tissue samples are minced to small pieces (~1mm^3) and transferred into a scraping device, while the bathing solution is changed to Ca^{2+}-free dissociation buffer containing (in mM): NaCl 113, KCl 4.7, KH_2PO_4 0.6, Na_2HPO_4 0.6, $MgSO_4$-$7H_2O$ 1.2, $NaHCO_3$ 12, $KHCO_3$ 10, HEPES 10, taurine 20, Na pyruvate 4, glucose 10, BDM 10 (pH 7.3 with NaOH) and heated to 37 ºC. Collagenase Type V and Protease Type XXIV (Sigma) were subsequently added and tissue chunks digested for a total 2 hours' time. During the digestion, the buffer containing dissociated myocytes was collected every 15 minutes from the scraping device and diluted with KB solution at room temperature. KB solution contained (in mM): KCl 20, KH2PO4 10, glucose 25, mannitol 5, L-glutamic acid monopotassium salt 70, β-hydroxybutyric acid 10, EGTA 10 and 2mg/mL albumin (pH 7.2 with KOH). The myocytes were left to settle and then resuspended in Ca^{2+}-free Tyrode solution containing (in mM): 132 NaCl, 4 KCl, 1.2 $MgCl_2$ 10 HEPES, and 11 glucose (pH 7.35 NaOH). $CaCl_2$ was added stepwise up to 0.6 mM. Cells were stored in this solution and used within 3 hours.

Quality control on cardiomyocytes. Cells are washed and transferred to a temperature-controlled recording chamber (experimental temperature= 35±0.5ºC), mounted on the stage of an inverted microscope, featuring electrodes for field stimulation. The selection of cells to be used for experiments is based on the following criteria: (i)well visible striations across the whole cardiomyocyte with uniform spacing, (ii) well-defined surface sarcolemma, (iii) absence of inclusions within the myocyte, (iv) absence of spontaneous contractions and (v) good contractile response to field stimulation (as shown in Supplementary Figure 1C-D). The fraction of cells where the aforementioned criteria are verified are approximately 20% in cell suspensions obtained from both HCM and control samples.

Current-clamp/intracellular Ca^{2+} studies on single myocytes: These experiments were conducted as we previously described[6]. Myocytes were incubated 30'with the Ca^{2+}indicator Fluoforte (Enzo Life Sciences, Farmingdale, New York) at room temperature, washed and transferred to the microscope-mounted recording chamber. Fluoforte fluorescence was detected at 505-520nm (using a high-speed high-sensitivity EMCCD Camera, model Evolve Delta by Photometrics, USA), during bright-field illumination at 492±3 nm (using a dedicated LED light source, model SpectraX by Lumencor). Experimental temperature was 35±5 °C for all protocols.

Intracellular Ca^{2+} was monitored during field stimulation at different pacing rates and under acute administration of drugs as detailed below. Action potentials (APs) were measured simultaneously using the perforated-patch configuration (amphotericin-B method). Specifically, for AP recordings, the pipette solution contained (in mM) 115 K methanesulfonate, 25 KCl, 10 HEPES, 3$MgCl_2$ and cells were superfused with Tyrode buffer (see above) containing 1.8mM $CaCl_2$. APs were elicited with short depolarizing stimuli (<3ms) at different frequency of stimulation (0.2Hz, 0.5Hz and 1Hz, 1 minute at each frequency).

EADs and DADs: EAD and DAD events were considered when a spontaneous depolarization larger than 20mV was detected during the plateau of an AP or during the diastolic period, respectively, while the cell is stimulated with the 3-minute protocol described above. A cardiomyocyte was scored positive for EADs or DADs if it displayed > 2 events during 3'of stimulated activity.

Drug studies: test recordings in presence of 5μM disopyramide were performed after >3 minutes from the beginning of drug exposure and repeated after >5minutes of washout.

Voltage-clamp studies: After cell stabilization, cardiomyocyte membrane capacitance was measured as previously described[6, 8]. Membrane current measurements were normalized by cell capacitance to calculate current densities.

Peak and Late sodium current ($I_{Na\text{-}peak}$ and I_{NaL}, respectively) were measured as detailed before[6,8]. Isolated ventricular cardiomyocytes were superfused with an extracellular solution containing (in mM) 135.0 NaCl, 4.6 CsCl, 1.1 $MgSO_4$, 10 glucose, 10.0 HEPES, and 0.01 nitrendipine, pH 7.4, at 25±1 °C. Pipette solution contained (in mM) 120 l-aspartic acid, 20 CsCl, 1 $MgSO_4$, 4 Na_2ATP, 0.1 Na_3GTP, 10 EGTA, 4.3 $CaCl_2$ (150 nM of calculated free Ca^{2+}) and 10 HEPES; pH 7.3 (with CsOH). To measure late $I_{Na\text{-}peak}$ and I_{NaL} and the extent of their block by disopyramide, 1-s depolarizing steps to −10 mV from a holding potential of −120 mV were applied to cells at a rate of 0.2 Hz: 10 subsequent episodes were averaged. The maximal amplitude of $I_{Na\text{-}peak}$ was measured in the first 10 seconds after the onset of depolarization. The magnitude of I_{NaL} was

determined by integration of the area of the current between 50 and 750ms after onset of the −10 mV clamp pulse, using the integration (area) feature of the pCLAMP program (Molecular Devices). Membrane current measurements were normalized by cell capacitance to calculate current densities.

L-Type Ca^{2+}-current was measured as previously described[6]. Isolated ventricular cardiomyocytes were superfused with an extracellular solution containing (in mM) 135.0 NaCl, 4.6 CsCl, 1.1 $MgSO_4$, 10 glucose, 10.0 HEPES, and 0.01 Tetrodotoxin, pH 7.4, at 36±1 °C. Pipette solution contained (in mM) 120 l-aspartic acid, 20 CsCl, 1 $MgSO_4$, 4 Na_2ATP, 0.1 Na_3GTP, 10 EGTA, 4.3 $CaCl_2$ (150 nM of calculated free Ca^{2+}) and 10 HEPES; pH 7.3 (with CsOH). 100 or 500 ms depolarizing steps to 0mV from a holding potential of -90mV were applied to the cell at a rate of 0.5Hz: 20 subsequent episodes were averaged. The measurements were repeated in the presence of disopyramide.

Delayer-rectifier K^+ currents were measured while perfusing the cell with a solution containing (in mM): 132 NaCl, 4 KCl, 1.2 $MgCl_2$, 1.8 $CaCl_2$, 10 HEPES, 11 glucose, 0.3 $CdCl_2$ and 30μM TTX (pH 7.35 NaOH); pipette solution contained(mM): K-l-Aspartic acid 130, HEPES 10, Na_2-ATP 5, Na_2-GTP 0.1, EGTA 11, $MgCl_2$ 2.0, $CaCl_2$ 5.0, pH adjusted to 7.2 with KOH. I_K recordings were carried out at 25°C by imposing 1s depolarization steps to a range of voltages from -40mV to +50 mV, starting from an holding potential of -80 mV. The current was measured at steady state (after> 500ms from the onset of depolarization). The measurements were repeated in the presence of disopyramide.

Signal recording and processing: Potential and current signals were measured with a Multiclamp 700B amplifier, using a standard 10kHz longpass digital filter. Patch-clamp signals were simultaneously digitized through using Digidata 1440A. Acquisition and analysis were controlled by dedicated software (pClamp10.0). All products from Molecular Devices, Sunnyvale, California. Ca-fluorescence videos were recorded at >200 frames per second and the fluorescence of single cells was analysed using Metafluor software (Molecular Devices). Absolute free intracellular Ca^{2+} concentrations $[Ca^{2+}]_i$ corresponding to Calcium dye Fluorescence values were estimated as previously done by Trafford et al. on ferret myocytes[9] and by Voigt et al. in human atrial cardiomyocytes[10].

Absolute free Ca^{2+} concentration ($[Ca^{2+}]_i$) corresponding to Fluoforte fluorescence (F) was calculated as follows:

$$[\mathrm{Ca}^{2+}]_i = \frac{K_d * F}{F_{max} - F}$$

K_d is the dissociation constant of Fluoforte (389 nmol/L), F is Fluoforte fluorescence, and F_{max} is Ca^{2+} saturated fluorescence obtained at the end of each experiment by damaging the cell with the

patch pipette. The K_d we used for Fluoforte (389 nmol/L) is the value published on the company's datasheet (www.enzolifesciences.com).
No filtering or post-hoc adjustment was added to the traces shown in this work.

Mechanical studies on intact trabeculae: These experiments were conducted as we previously described[6]. Intact ventricular trabeculae were mounted between a basket-shaped end of a force transducer (KG7A, Scientific Instruments Heidelberg, Germany) and a motor (Aurora Scientific Inc., Aurora, Canada), controlled by a custom Labview (National Instruments, Austin, Texas) program. Muscles were mounted in cold cardioplegic solution and then perfused with Krebs/Henseleit buffer, containing (in mM) 119 NaCl, 4.7 KCl, 2.5 $CaCl_2$, 1.2 $MgSO_4$, 1.2 KH_2PO_4, 25 $NaHCO_3$; pH 7.4 with 95%O_2:5%CO_2. Muscles were allowed to stabilize for at least 30 min before recordings and muscle length was empirically adjust to "optimal length" as previously described. Briefly, slack muscles were progressively stretched while monitoring the increase in the development of active tension. Resting tension remained negligible during the initial stretch phase. To reach the optimal sarcomere length, muscles were stretched step-by-step (2% of muscle length per step) to a length where a small increase in length resulted in no further increases in active tension, while resting tension started to rise[11]. This "optimal length" was selected to be comparable to the maximally attained length *in vivo* (2.15±0.05 μm sarcomere length)[12].

Isometric force was recorded at 35±2°C under various conditions including various pacing rates (0.1-2.5 Hz) and acute drugs administration (below). The occurrence of premature beats was evaluated during steady state stimulation (0.5 Hz) ad during stimulation pauses. Specifically, one-minute stimulation pauses were inserted after the last contraction of a steady series at 3 Hz to promote the occurrence of the spontaneous contractions. At the end of each experiment, muscle section was measured for force normalization.

Drugs tests**:** We tested the effects of Disopyramide at 5 μM, unless otherwise specified. Test recordings in presence of the drug were performed after >3 minutes from the beginning of drug exposure.

Mechanical studies on skinned trabeculae: In order to assess myofilament function, muscle strips and trabeculae were skinned by overnight incubation in relaxing solution added with 0.5% Triton X100. Of note, trabeculae used for skinned muscle studies were taken from the same patient samples that were used for the other experiments; however, these were not the same trabeculae that were employed for intact muscle studies (see above). Triton was then removed and the skinned preparations were mounted horizontally between a force transducer and a motor by means of T-clips.

Muscles were activated by transferring them manually between baths containing different pCa solutions and the pCa-force relationship was determined. Three types of solutions were employed: relaxing solution (pCa 9) with 5 mM EGTA, pre-activating solution with 0.5 mM EGTA and 4.5 mM 1,6-diamino hexane-N,N,N',N'-tetraacetic acid (HDTA), and a maximal activating (pCa 4.5) solution with 5 mM CaEGTA. Relaxing and maximal activating solutions were mixed in different proportions to obtain activating solutions with various pCa's. Solutions were applied in the sequence: relaxing, pre-activating, activating, relaxing. All solutions contained: 60 mM BES (N,N-bis[2-hydroxyethyl]-2-aminoethane sulphonic acid); 5.83 mM Na_2ATP_2, 7.4 mM MgCl. Potassium propionate was added to adjust the final ionic strength to 0.20 M. pH was adjust to 7.1 with KOH at 20°C.

Sarcomere energetics in skinned trabeculae. The experimental procedures, solutions and equipment used for functional measurements were as described previously [13, 14]. Isometric force and ATPase activity were measured at maximal and submaximal [Ca^{2+}] at 20°C. During the measurement trabeculae were kept inside a small (30 µl) chamber with thin quartz windows.

ATPase activity was measured using an enzyme coupled assay. In this assay the ATP regeneration from ADP and phosphoenol-pyruvate, catalyzed by the enzyme pyruvate kinase, is coupled to the oxidation of NADH to NAD+ and the reduction of pyruvate to lactate, catalyzed by L-lactic dehydrogenase. NADH oxidation was measured photometrically from the absorbance at 340 nm of near-UV light. The absorbance signal was calibrated by adding 0.5 nmol ADP to the solution in the measuring chamber. ATPase activity in the muscle could be derived from the slope of the absorbance signal. The Ca^{2+}-activated ATPase activity was calculated by subtracting the basal ATPase activity (measured in relaxing solution) from the NADH oxidation during contraction and normalized to the volume of the trabeculae. The tension cost (energy cost of tension generation) is expressed as the ratio ATPase activity/tension).

Ca^{2+} sparks in permeabilized myocytes: Left ventricular cardiomyocytes were isolated from the hearts of 4 transgenic HCM mice carrying the R92Q troponin-T mutation, as previously described[15, 16]. Cells were exposed for 90 s to saponin (0.01%) added to an internal solution containing (mmol/L): 120 K-aspartate, 10 HEPES, 3 MgATP, 0.5 EGTA, 10 Na phosphocreatine, 5 U/mL creatine phosphokinase, 0.75 $MgCl_2$ and 8% dextran, pH 7.2 (intracellular buffer) [17]. After permeabilization, cells were resuspended in an intracellular buffer (as above) containing 150nM free [Ca^{2+}] and 5µM of the Ca^{2+}-sensitive dye Asante Na^+-green K^+-salt (Teflabs, USA). The concentration of free Ca2+ calculated with Maxchelator 2 (http://maxchelator.stanford.edu/). Ca^{2+} sparks were recorded in cells perfused with the intracellular buffer in the absence of saponin. Images were obtained with confocal microscopy (Nikon, objective o.i. 63x, n.a. 1.4) by scanning the cell with

an Argon laser along the longitudinal cell axis[17] every 1.95 ms; fluorescence was excited at 488 nm and emissions were collected at >505 nm. Image analyses were performed by homemade routines using ImageJ. Images were corrected for the background fluorescence. The fluorescence values (F) were normalized by the basal fluorescence (F0) in order to obtain the fluorescence ratio (F/F0). Ca^{2+} sparks were detected using an automated detection system and a criterion that limited the detection of false events while detecting most Ca^{2+} sparks, using the SparkMaster ImageJ plugin for automated analysis[18].

Chemicals: unless otherwise specified, all chemicals were purchased from Sigma-Aldrich, St. Louis, MO.

Statistical analysis for basic studies. Statistical analysis was performed as we previously described[6]. Data from studies on isolated myocytes and trabeculae are expressed and plotted as means±SEM (Standard Error of Mean) values obtained from a number of independent determinations on different myocytes or muscles: number of cells/trabeculae and number (and ID#) of patients are indicated in the figure legends for each set of measurements.

To faithfully compare different sets of measurements, sensitivity analysis was performed for each statistical comparison, in order to account for:

1- non Gaussian distribution

The data were tested for normality using the Skewness/Kurtosis test[19].

Non-parametric test based on rank transformation (Wilcoxon's sum of rank) was used to check robustness of results if the data were not normally distributed.

2- heteroschedasticity (inequality of variances)

We used the F test for equality of variances in two-group comparison studies and the Bartlett's test for variance homogeneity in the multiple comparison design.

.

3- within-subject correlation

Most of the average data derives from multiple myocytes or trabecuale from different patients. We estimated within-subject correlation for each variable with repeated measures with One-way ANOVA. In order to account for the correlation among different cells/muscles from the same patient, we used linear mixed models[20] to compare couples of data groups. In order to reduce the risk of type I errors resulting from the stronger interrelationship among cells/trabeculae isolated from the same patient sample, we used hierarchical statistics including two nested levels (patients and

cells/trabeculae)(22), plus a third hierarchical level (presence or absence of disopyramide in the same cell/trabecula) to assess the effects of drug treatment in a pairwise fashion.

This approach was implemented using linear mixed models in Stata 12.0 (StataCorp LLC, USA). In particular, we used the following command in Stata 12.0.

```
. xi: xtmixed var diso  ||  subj:   || cellno:
```

Where "var" is the experimental variable, "diso" is 0 or 1 depending on the absence or presence of disopyramide, "subj" is patient sample ID and "cellno" is the unique number of the cell or trabecula from which each measurement was performed .

Correction for heteroschedasticity was applied to linear mixed models in unpaired comparisons whenever the variances of the two groups were unequal (as calculated by F-test).

All the results of the new statistical analysis are available on request. The Probability (P) values that are shown in the manuscript and in the online supplement were calculated with linear mixed models according to the aforementioned procedure, both when comparing repeated measurements on the same samples (e.g. effect of disopyramide) and when comparing unpaired datasets. P-values <0.05 were considered statistically significant.

To quantify EADs/DADs and spontaneous contractions occurrence, including their confidence intervals, the binomial proportion confidence interval was calculated as approximating the binomial distribution with a normal distribution (central limit theorem). The confidence interval was calculated as:

$$\hat{p} \pm \sqrt{\frac{\hat{p}(1-\hat{p})}{N}}$$

where $\hat{p}$ is the fraction of successes in a Bernoulli trial process estimated from the statistical sample and N is the sample size. In our case $\hat{p}$ is the fraction of myocytes scored positive for EAD or DAD events as described above.

The central limit theorem was not applied to a binomial distribution where the fraction $\hat{p} \approx 0$. In these cases the Wilson score interval was applied:

$$\frac{\left(\hat{p} + \frac{1}{2N}\right) \pm \sqrt{\frac{\hat{p}(1-\hat{p})}{N} + \frac{1}{4N^2}}}{1 + \frac{1}{N}}$$

The statistical significance of differences in DAD or EAD occurrence was assessed using the Fisher exact test. Statistical analysis was performed using Stata 12 software (StataCorp LP, College Station, Texas, USA).

Modelling studies: Cellular mechanisms of Diso action on dispersion of repolarization were investigated in a population of human ventricular cardiomyocyte models (n=250), in control vs HCM remodelling conditions[21]. In brief, the population was based on the O'Hara-Rudy model of the human cardiac ventricular action potential[22], accounting for variability in all main inward and outward ion channel currents, as well as in intracellular processes regulating Ca^{2+} handling. For each model in the population, its HCM counterpart was generated by including the human-specific HCM remodelling described in Coppini et al, 2013[6]. Only models satisfying human AP and Ca^{2+} transient biomarkers, in both control and HCM conditions, were retained in the final population. The action of 5 μM Diso was modelled based on the characterization of drug action performed in this study, by incorporating reductions of 22% I_{Na}, 45% I_{NaL}, 16% $I_{Ca\text{-}L}$, 15% I_{Kr}, and a 50% RyR inhibition in order to match the observed reduction of Ca^{2+}-transient amplitude. All cell models were paced at 1 Hz for a total of 500 beats, which was sufficient to obtain stable AP and Ca^{2+}-transient biomarkers.

Mechanisms of dispersion at the whole ventricular level were investigated in an MRI-based anatomical model of an obstructive HCM patient, under realistic human activation sequence and heterogeneity in repolarizing currents, as described in Lyon et al, Europace 2018[23]. A corresponding torso mesh was also included in the simulations, with virtual electrodes placed on standard positions to simulate the 12-leads ECG[23, 24].

At the whole-organ level, our data from obstructive HCM patients supports the presence of ionic remodeling in regions of marked hypertrophy. Previous modeling studies also showed that such co-localization was the only mechanism that could recapitulate T-wave inversion in HCM patients with apical hypertrophy[23]. In the ventricular mesh, the representative control and HCM models illustrated in Figure 6A were assigned to the non-hypertrophic and hypertrophic regions, respectively, with steady-state values obtained after simulating 500 beats at 1 Hz pacing, and a 30% and 70% transmural split ratio of epicardial to endocardial tissue. The action of 5 μM Diso was modelled as described above, together with a reduction of 22% in conduction velocity in the fast endocardial activation layer used to mimic the sub-endocardial Purkinje network[24], in order to reflect the changes in sodium current dynamics due to disopyramide application. For each case, two consecutive heart beats were simulated in sinus rhythm at 1 Hz pacing, producing consistent activation and repolarization maps and consistent ECG traces in all leads.

REFERENCES:

1. Ball W, Ivanov J, Rakowski H, Wigle ED, Linghorne M, Ralph-Edwards A, Williams WG, Schwartz L, Guttman A and Woo A. Long-term survival in patients with resting obstructive

hypertrophic cardiomyopathy comparison of conservative versus invasive treatment. *Journal of the American College of Cardiology*. 2011;58:2313-21.
2. Sherrid MV, Barac I, McKenna WJ, Elliott PM, Dickie S, Chojnowska L, Casey S and Maron BJ. Multicenter study of the efficacy and safety of disopyramide in obstructive hypertrophic cardiomyopathy. *Journal of the American College of Cardiology*. 2005;45:1251-8.
3. Sherrid MV, Shetty A, Winson G, Kim B, Musat D, Alviar CL, Homel P, Balaram SK and Swistel DG. Treatment of obstructive hypertrophic cardiomyopathy symptoms and gradient resistant to first-line therapy with beta-blockade or verapamil. *Circulation Heart failure*. 2013;6:694-702.
4. Adler A, Fourey D, Weissler-Snir A, Hindieh W, Chan RH, Gollob MH and Rakowski H. Safety of Outpatient Initiation of Disopyramide for Obstructive Hypertrophic Cardiomyopathy Patients. *Journal of the American Heart Association*. 2017;6.
5. Sherrid MV and Arabadjian M. A primer of disopyramide treatment of obstructive hypertrophic cardiomyopathy. *Progress in cardiovascular diseases*. 2012;54:483-92.
6. Coppini R, Ferrantini C, Yao L, Fan P, Del Lungo M, Stillitano F, Sartiani L, Tosi B, Suffredini S, Tesi C, Yacoub M, Olivotto I, Belardinelli L, Poggesi C, Cerbai E and Mugelli A. Late sodium current inhibition reverses electromechanical dysfunction in human hypertrophic cardiomyopathy. *Circulation*. 2013;127:575-84.
7. Coppini R, Ferrantini C, Aiazzi A, Mazzoni L, Sartiani L, Mugelli A, Poggesi C and Cerbai E. Isolation and functional characterization of human ventricular cardiomyocytes from fresh surgical samples. *Journal of visualized experiments : JoVE*. 2014.
8. Undrovinas NA, Maltsev VA, Belardinelli L, Sabbah HN and Undrovinas A. Late sodium current contributes to diastolic cell Ca2+ accumulation in chronic heart failure. *The journal of physiological sciences : JPS*. 2010;60:245-57.
9. Trafford AW, Diaz ME and Eisner DA. A novel, rapid and reversible method to measure Ca buffering and time-course of total sarcoplasmic reticulum Ca content in cardiac ventricular myocytes. *Pflugers Archiv : European journal of physiology*. 1999;437:501-3.
10. Voigt N, Li N, Wang Q, Wang W, Trafford AW, Abu-Taha I, Sun Q, Wieland T, Ravens U, Nattel S, Wehrens XH and Dobrev D. Enhanced sarcoplasmic reticulum Ca2+ leak and increased Na+-Ca2+ exchanger function underlie delayed afterdepolarizations in patients with chronic atrial fibrillation. *Circulation*. 2012;125:2059-70.
11. Rodriguez EK, Hunter WC, Royce MJ, Leppo MK, Douglas AS and Weisman HF. A method to reconstruct myocardial sarcomere lengths and orientations at transmural sites in beating canine hearts. *The American journal of physiology*. 1992;263:H293-306.
12. Ross J, Jr., Sonnenblick EH, Taylor RR, Spotnitz HM and Covell JW. Diastolic geometry and sarcomere lengths in the chronically dilated canine left ventricle. *Circulation research*. 1971;28:49-61.
13. Witjas-Paalberends ER, Ferrara C, Scellini B, Piroddi N, Montag J, Tesi C, Stienen GJ, Michels M, Ho CY, Kraft T, Poggesi C and van der Velden J. Faster cross-bridge detachment and increased tension cost in human hypertrophic cardiomyopathy with the R403Q MYH7 mutation. *The Journal of physiology*. 2014;592:3257-72.
14. Witjas-Paalberends ER, Guclu A, Germans T, Knaapen P, Harms HJ, Vermeer AM, Christiaans I, Wilde AA, Dos Remedios C, Lammertsma AA, van Rossum AC, Stienen GJ, van Slegtenhorst M, Schinkel AF, Michels M, Ho CY, Poggesi C and van der Velden J. Gene-specific increase in the energetic cost of contraction in hypertrophic cardiomyopathy caused by thick filament mutations. *Cardiovascular research*. 2014;103:248-57.
15. Coppini R, Mazzoni L, Ferrantini C, Gentile F, Pioner JM, Laurino T, Santini L, Bargelli V, Rotellini M, Bartolucci G, Crocini C, Sacconi L, Tesi C, Belardinelli L, Tardiff J, Mugelli A, Olivotto I, Cerbai E and Poggesi C. Ranolazine Prevents Phenotype Development in a Mouse Model of Hypertrophic Cardiomyopathy. *Circulation Heart failure*. 2017;10.
16. Ferrantini C, Coppini R, Pioner JM, Gentile F, Tosi B, Mazzoni L, Scellini B, Piroddi N, Laurino A, Santini L, Spinelli V, Sacconi L, De Tombe P, Moore R, Tardiff J, Mugelli A, Olivotto I,

Cerbai E, Tesi C and Poggesi C. Pathogenesis of Hypertrophic Cardiomyopathy is Mutation Rather Than Disease Specific: A Comparison of the Cardiac Troponin T E163R and R92Q Mouse Models. *Journal of the American Heart Association*. 2017;6.
17. Fernandez-Velasco M, Rueda A, Rizzi N, Benitah JP, Colombi B, Napolitano C, Priori SG, Richard S and Gomez AM. Increased Ca2+ sensitivity of the ryanodine receptor mutant RyR2R4496C underlies catecholaminergic polymorphic ventricular tachycardia. *Circulation research*. 2009;104:201-9, 12p following 209.
18. Picht E, Zima AV, Blatter LA and Bers DM. SparkMaster: automated calcium spark analysis with ImageJ. *American journal of physiology Cell physiology*. 2007;293:C1073-81.
19. Sullivan LM and D'Agostino RB. Robustness and power of analysis of covariance applied to data distorted from normality by floor effects: homogeneous regression slopes. *Statistics in medicine*. 1996;15:477-96.
20. Rabe-Hesketh S, Skrondal, A. and Pickles, A. Generalized multilevel structural equation modelling. . *Psychometrika*. 2004;69 167-190.
21. Passini E, Minchole A, Coppini R, Cerbai E, Rodriguez B, Severi S and Bueno-Orovio A. Mechanisms of pro-arrhythmic abnormalities in ventricular repolarisation and anti-arrhythmic therapies in human hypertrophic cardiomyopathy. *Journal of molecular and cellular cardiology*. 2016;96:72-81.
22. O'Hara T, Virag L, Varro A and Rudy Y. Simulation of the undiseased human cardiac ventricular action potential: model formulation and experimental validation. *PLoS computational biology*. 2011;7:e1002061.
23. Lyon A, Bueno-Orovio A, Zacur E, Ariga R, Grau V, Neubauer S, Watkins H, Rodriguez B and Minchole A. Electrocardiogram phenotypes in hypertrophic cardiomyopathy caused by distinct mechanisms: apico-basal repolarization gradients vs. Purkinje-myocardial coupling abnormalities. *Europace* 2018;20:iii102-iii112.
24. Cardone-Noott L, Bueno-Orovio A, Minchole A, Zemzemi N and Rodriguez B. Human ventricular activation sequence and the simulation of the electrocardiographic QRS complex and its variability in healthy and intraventricular block conditions. *Europace*2016;18:iv4-iv15.

Supplementary Table 1

**HCM Patients for *in vitro* studies (n=20)**	
Age at surgery	51 ± 7 yrs
Gender	Female 9/20 (45%)
NYHA Class II	12/20
NYHA Class III	8/20
Genotype	12/20 (7 *MYBPC3*, 3 *MYH7*, 2 SARCOMERE-NEG.)
**Arrhythmic risk**	
Syncope	7/20 (33%)
Non-sustained ventric. tachycardia	10/20 (50%)
History of Atrial Fibrillation	9/20 (45%)
ICD implanted	4/20 (20%)
**Echo features**	
Maximal septal thickness	26±5 mm
Ejection fraction	66 ± 4 %
LVOT grad. at rest >30mmHg	20/20 (100%)
LVOT gradient at rest	78 ± 7 mmHg
LA end-systolic volume	109 ± 39 mL
Severe diastolic dysfunction (pseudonormalized,restrictive)	10/20 (50%)
**Pharmacological Therapy**	
Beta blockers	20/20 (100%)
Disopyramide	10/20 (50%)
Amiodarone	2/20 (10%)
Diuretics/ACE-Inhibitors	10/20 (50%)
**Known Comorbidities**	
Coronary artery disease	0/20 (0%)
Diabetes	0/20 (0%)
Renal and/or hepatic dysfunction	0/20 (0%)

Supplementary Table 1: Clinical features of the HCM patients included in the in vitro study. Clinical data refers to visits performed less than 1 month before the myectomy operation. Categorical data is expressed as proportion of patients; continuous values are expressed as mean ± standard deviation (SD). NYHA= New York Heart Association; MYPBC= Myosin-Binding Protein C; MYH= Myosin Heavy Chain; ICD= Implantable Cardioverter Defibrillator; LVOT= Left-Ventricular Outflow Tract; LA= Left Atrium.

Supplementary Table 2

Control Patients (n=4)	
Age at surgery	57 ± 8 yrs
Gender	Female 2/4 (50%)
NYHA Class I	4/4 (100%)
NYHA Class II	0/4 (0%)
**Arrhythmic risk**	
Syncope	0/4 (0%)
Non-sustained ventric. tachycardia	0/4 (0%)
History of Atrial Fibrillation	0/4 (0%)
**Echo features**	
Maximal septal thickness	12±2 mm
Ejection fraction	59 ± 6 %
LVOT grad. at rest >30mmHg	0/4 (0%)
Bulging septum	4/4 (100%)
LA end-systolic volume	81 ± 16 mL
Severe diastolic dysfunction (pseudonormalized,restrictive)	0/4 (0%)
**Pharmacological Therapy**	
Beta blockers	1/4 (25%)
Disopyramide	0/4 (0%)
Amiodarone	0/4 (0%)
Diuretics/ACE-Inhibitors	0/4 (0%)
**Reason for surgery**	
Aortic valve insufficiency	3/4 (75%)
Severe mitral prolapse	1/4 (25%)
**Known Comorbidities**	
Coronary artery disease	0/4 (0%)
Diabetes	0/4 (0%)
Renal and/or hepatic dysfunction	0/4 (0%)

Supplementary Table 2: Control Patients Characteristics. Clinical features of the control patients enrolled in the study. Clinical data refers to visits performed less than 1 month before the operation. Categorical data is expressed as proportion of patients; continuous values are expressed as mean ± standard deviation (SD). NYHA= New York Heart Association; LVOT= Left-Ventricular Outflow Tract; LA= Left Atrium.

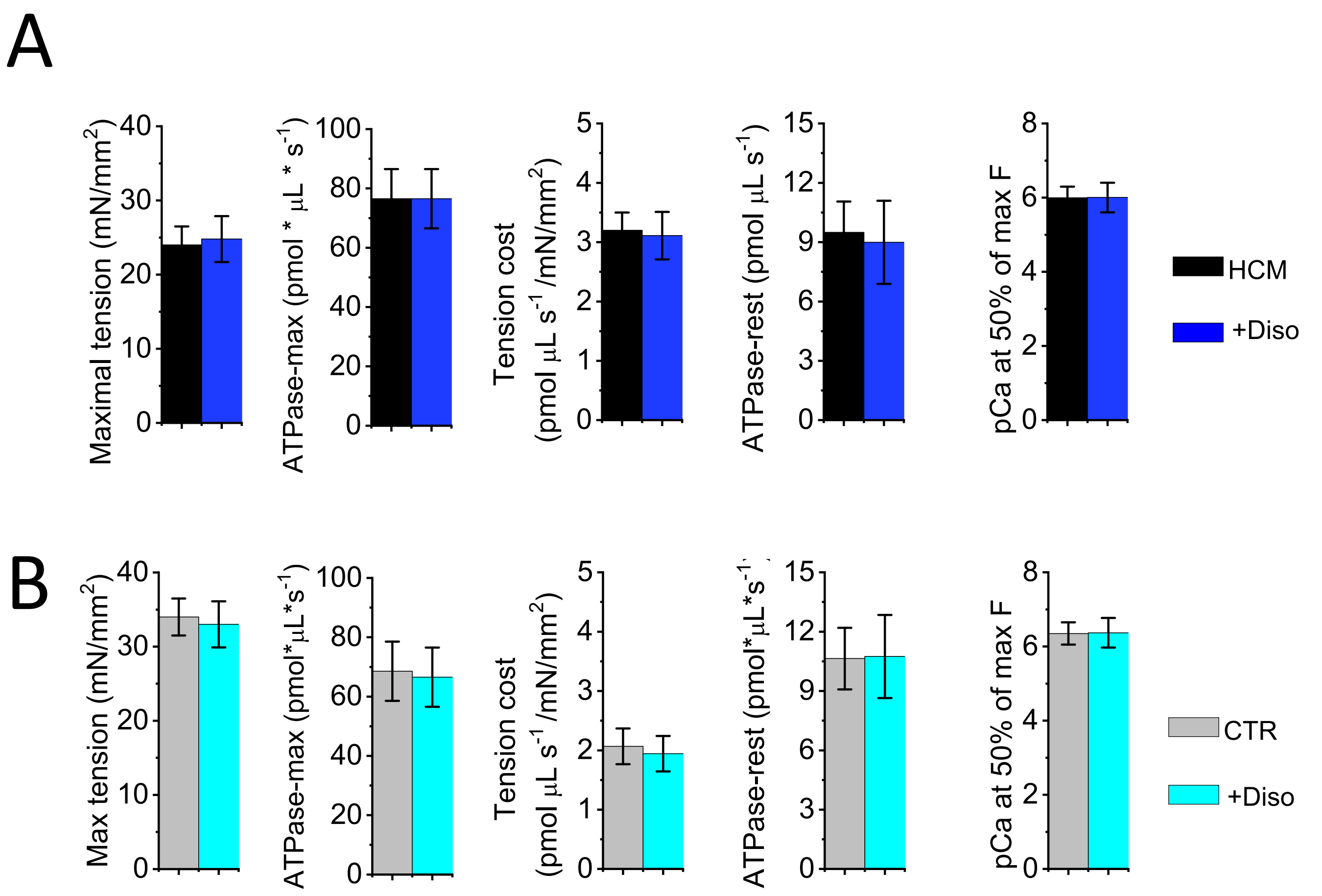

Supplemental Figure 1: Diso has no effects in HCM and control demembranated trabeculae. (A) Effects of Diso on the mechanical and energetic properties of HCM demembranated trabeculae (black columns= baseline; blue columns= 5μm Diso). From left to right: (i) maximal developed tension, (ii) maximal ATPase activity, as assessed during the development of maximal tension, (iii) energy cost of tension generation, calculated from the ratio of max. ATPase and max. tension; (iv) ATP consumption at rest (low [Ca]); (v) pCa at half-maximal force (calcium sensitivity of myofilaments). Means ± S.E.M. from 6 trabeculae, 3 HCM patients. **(B)** Effects of Diso on the mechanical and energetic properties of control demembranated trabeculae (grey columns= baseline; cyan columns= 5μm Diso). The bar graphs show the same parameters as in A. Means ± SEM from 4 trabeculae, 2 CTR patients.

Supplementary Figure 2

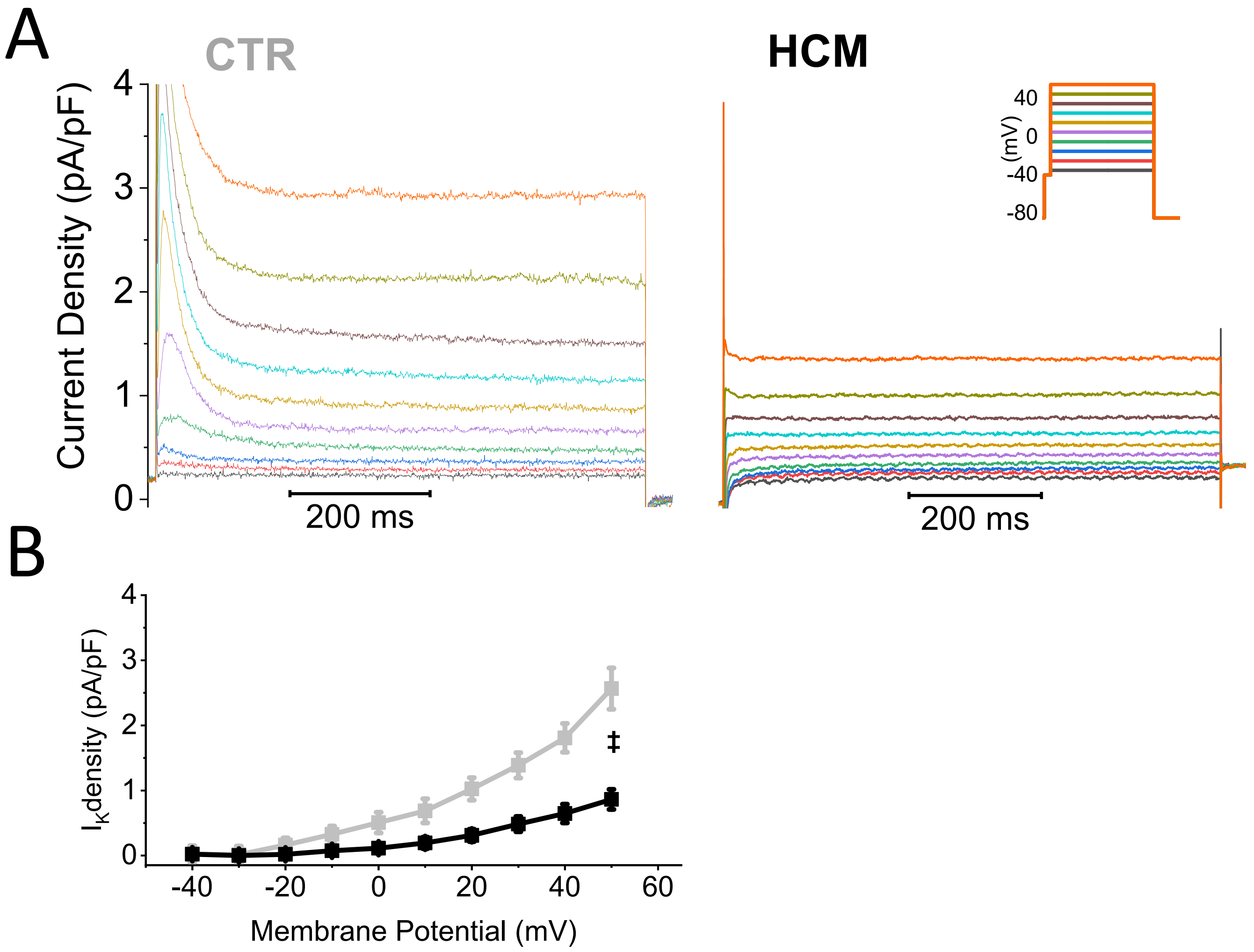

Supplemental Figure 2: Delayed rectifier potassium currents in HCM vs. control cardiomyocytes. (A) Representative traces of inward-rectifier potassium currents elicited at different voltages (see inlet panel for color codes) in a control cardiomyocyte (left, CTR) and an HCM cell (right). Notably, the early peak outward current (corresponding to transient outward current, I_{to}) is absent in HCM myocytes, as previously shown in Coppini et al. Circulation 2013. **(B)** Average I_K current density in control and HCM cardiomyocytes at different voltages. Means ± SEM from 8 cardiomyocytes, 3 patients (CTR) and 10 cardiomyocytes, 4 patients (HCM). ‡=P<0.05 for voltages ≥-10mV, linear mixed models.

Supplementary Figure 3

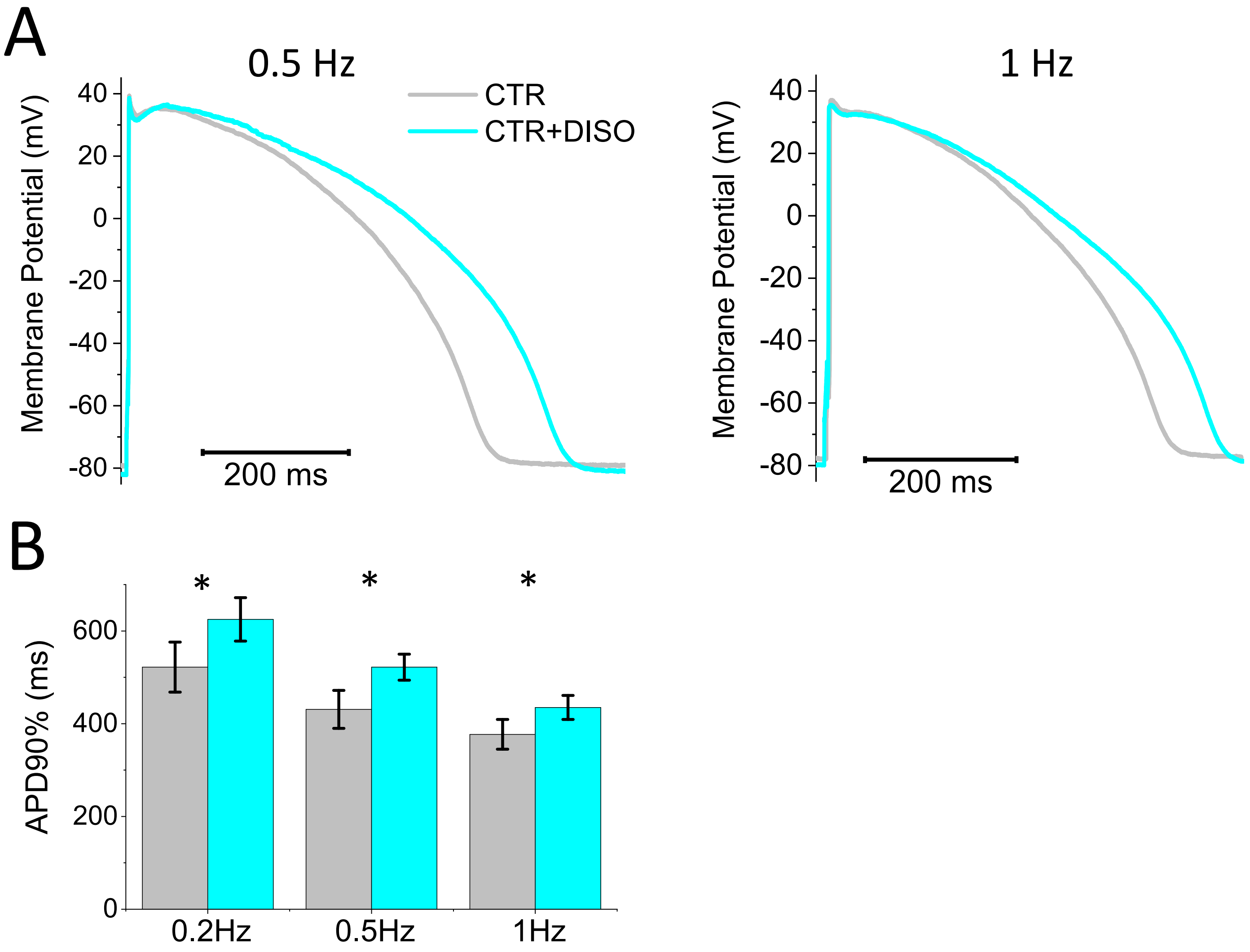

Supplemental Figure 3: Effects of disopyramide on action potentials in control cardiomyocytes. **(A)** Representative superimposed action potentials at baseline (grey traces) and in the presence of disopyramide 5µM (Diso, cyan traces), elicited at 0.5 Hz (left) and at 1 Hz (right), in a control cardiomyocyte. **(B)** Action potential duration at 90% of repolarization (APD90%) at baseline (grey) and in the presence of Diso (cyan) in control myocytes, at the different pacing rates. Means ± SEM from 9 myocytes, 2 patients. *P<0.05, linear mixed models

Supplementary Figure 4

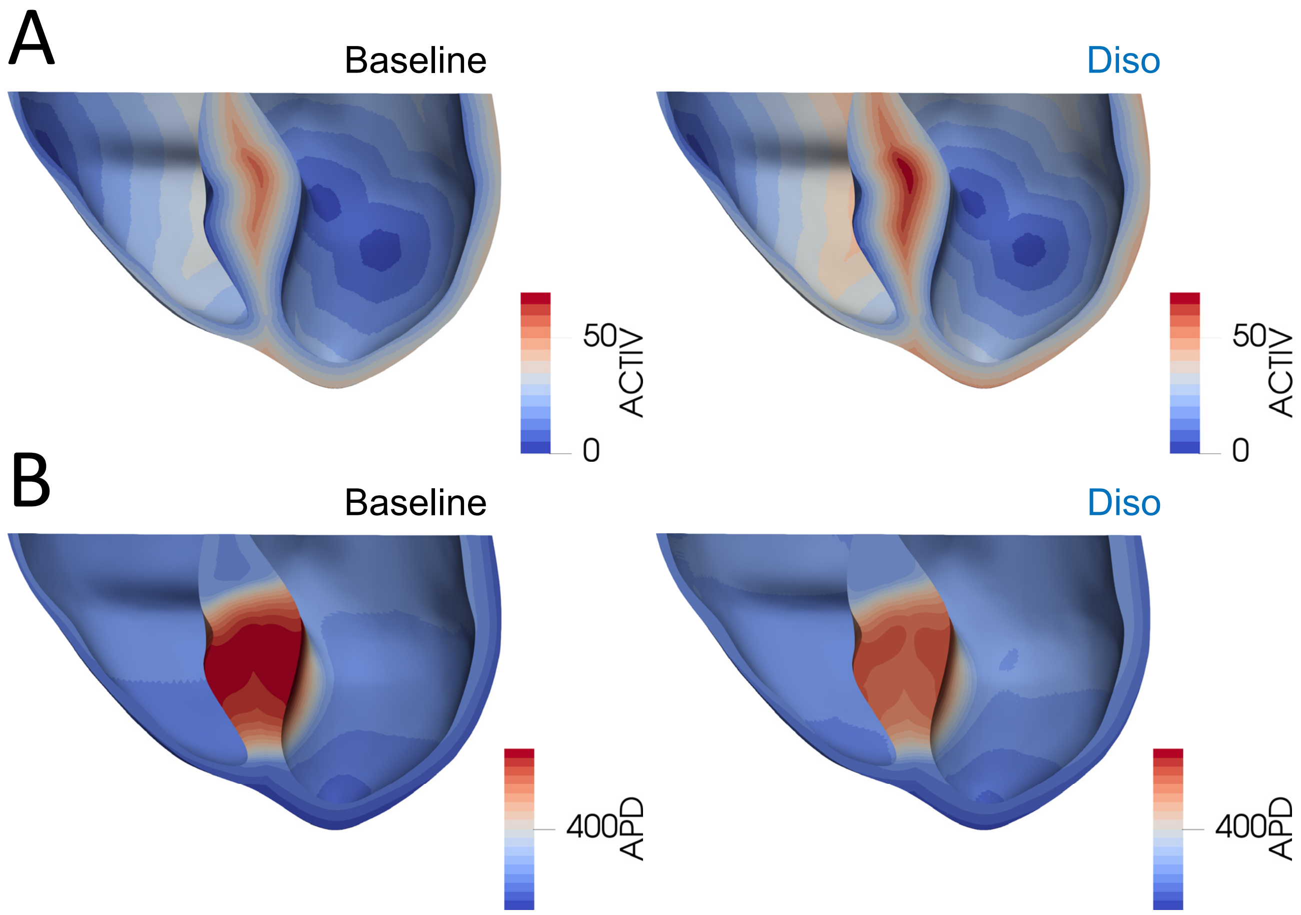

Supplemental Figure 4: Modelling results on disopyramide action on ventricular activation times and action potential duration. (A) Activation time maps show a reduced conduction velocity of action potential propagation after disopyramide application, consistent with its Class I antiarrhythmic profile. In baseline, total activation time was 61 ms, increased to 72 ms (11 ms increase) due to disopyramide action. **(B)** Action potential duration maps at 90% repolarization. In baseline, the mean and standard deviation of APD90% in the hypertrophic region were 500±60 ms, with a minimum-to-maximum range of 285 ms (340 to 625 ms). Under disopyramide, the mean and standard deviation were 469.4±47.3 ms, with a minimum-to-maximum range of 218 ms (318 to 536 ms). Disopyramide has the effect of reducing the range of APD90% in the hypertrophic region by 67 ms. In the whole biventricular mesh, APD90% were 318.7±89.2 ms in baseline, with a minimum-to-maximum range of 356 ms (223 ms to 579 ms), whereas under disopyramide the APD90% distribution was 322.0±73.1 ms, with a minimum-to-maximum range of 303 ms (233 ms to 536 ms). These statistics further show that disopyramide has the effect of reducing the range of APDs by 53 ms in the biventricular heart, thus highlighting the potential arrhythmo-protective effect of the drug.
